# Antioxidant Properties of the Vam3 Derivative of Resveratrol

**DOI:** 10.3390/molecules23102446

**Published:** 2018-09-25

**Authors:** Seyedmohammad Ahmadi, Tiziana Marino, Mario Prejanò, Nino Russo, Marirosa Toscano

**Affiliations:** 1 Department of Food Science and Technology, Faculty of Agriculture, University of Zabol, P.O. Box 98615-538, Zabol 98613-35856, Iran; sma_257@yahoo.com; 2 Dipartimento di Chimica e Tecnologie Chimiche, Università della Calabria, Cubo 14C, Via P. Bucci, 87036 Arcavacata di Rende, CS, Italy; tiziana.marino65@unical.it (T.M.); mario.prejano@unical.it (M.P.); nrusso@unical.it (N.R.)

**Keywords:** antioxidants, density functional theory, Vam3, resveratrol derivative

## Abstract

A considerable number of studies has shown that many constituents of foods derived from plants are effective and safe antioxidants. This explains the growing interest in natural antioxidants in food applications. The goal of this investigation was to evaluate the antioxidant properties of the Vam3, a resveratrol derivative, firstly isolated from ethanol extracts of *Vitis amurensis Rupr* as a secondary product, and to carry out a comparison with resveratrol and other phenolic compounds which are currently in the limelight all over the world due to their beneficial effects on the human body. The potential of Vam3 as an antioxidant was determined through the evaluation of some key thermodynamic parameters which are commonly used for this purpose and describe the antioxidant activity quite well. Various mechanisms through which antioxidants usually can carry out their action were also explored both in water and in apolar environment. The results indicated that Vam3 is an excellent candidate as a natural antioxidant.

## 1. Introduction

Free radicals are particularly reactive and unstable molecular entities that can form within our body when a closed-shell molecule, subjected to the stress of radiation and harmful agents, loses or gains an electron or undergoes a reaction such as hydrogen loss. The molecule, now unstable, tries to cling to the cellular structures around itself, in an attempt to recover its stability. When reacting with structures like lipids, proteins and DNA, free radical can generate another radical triggering a chain reaction that can lead to their damage. At the right concentrations and under the right conditions, free radicals are essential for the normal physiological processes of the organism [1,2,3,4]. In the presence of an accumulation of free radicals we talk instead of oxidative stress. This will cause injury to a large number of biomolecules with the formation of compounds that can lead to severe dysfunction of the cells that in turn can contribute to the genesis and development of a large number of diseases [1,2,3,4]. However, cells have a real army of molecules ready to counteract the harmful effects deriving from an excess of reactive species. The substances capable of interfering with chemical oxidation reactions that give rise to free radicals or neutralize those already produced are called antioxidants. Very different compounds belong to this category, either naturally present in the organism (endogenous) or ingested with food. The antioxidants that are taken from the outside to reinforce the cellular systems and defend them from free radicals include some vitamins, plant polyphenols and some trace elements essential for the functioning of the body′s antioxidant enzymes (selenium, copper, manganese and zinc).

Many foods and beverages of vegetable origin contain several nonflavonoid classes of phenolic compounds derived from plants [5]. Among them, resveratrol has been identified as the major active compound of stilbene phytoalexins and is presumed to be beneficial for human health in that it seems to lower cholesterol levels in the blood, stimulate the production of collagen and give tone and elasticity to the skin. Resveratrol can be extracted from the plant *Polygonum cuspidatum*, from peanuts, mulberries, from the grape or from the wine [6]. However, it is the *Polygonum*, which contains the highest concentration of resveratrol in the roots. Much has been discussed about resveratrol role in the so-called “French paradox” [7,8], the phenomenon whereby in France, despite the high consumption of foods rich in saturated fatty acids, the incidence of mortality for cardiovascular disease is lower than in other countries with a similar diet. Hence the association between the consumption of red wine and the apparent low incidence of heart disease, a correlation that has always been very criticized for lack of sufficient scientific evidence [9]. However, many studies indicated benefits of resveratrol in slowing down ageing and maintaining healthy cells recommending it as antioxidant to prevent cardiovascular disease [8,10,11,12,13].

Still today, there is great interest on natural and safer antioxidants in food applications, and a growing trend in consumer preferences. This justify the continuous impetus to explore natural sources of antioxidants.

**Figure 1:**
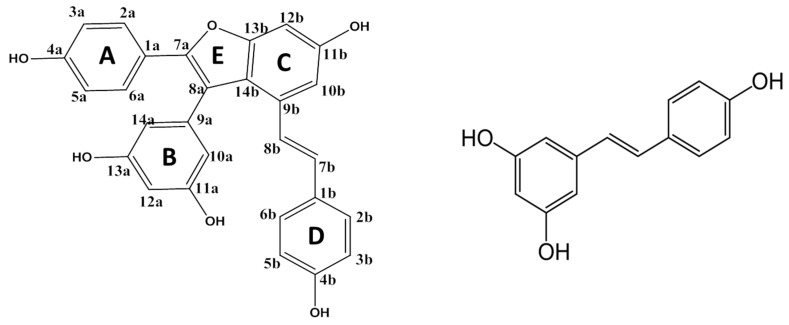
Schematic structures of Vam3 (**left**) and resveratrol (**right**).

Vam3 (Figure 1) is a derivative of resveratrol which was first isolated as a secondary natural product from ethanol extracts of *Vitis amurensis Rupr* which grows in northeastern and central China. Previous studies showed that Vam3 possesses potent anti-inflammatory effects and is able to inhibit Syk kinase in mast cell [14,15]. The role of Vam3 as an antioxidant has never been explored, despite the similarity of this compound with a significant portion of the resveratrol molecule (Figure 1), which suggests that it could be a good antioxidant candidate. Vam3 exhibits the typical features which constitute the structural basis for the antioxidant activity of polyphenolic systems, that is, the concomitant presence of many hydroxyl groups and the planarity of a significant part of the molecule which allow the formation of several radical species that can be stabilized through resonance, delocalization and conjugation effects (see Figure 1).

On the other hand, the fact that C and D rings of Vam3 share the same structure with resveratrol was exploited as an inspiring principle behind a study that demonstrated that Vam3, like resveratrol, inhibits Syk kinase in mast cell [15].

As in our previous theoretical studies on similar systems [16,17,18,19,20], and also in the present work, we have investigated the most common mechanisms used by antioxidants when they perform their action against free radicals. These mechanisms, explored making use of the density functional theory (DFT)-based methods, are usually referred as hydrogen atom transfer (HAT), single electron transfer—proton transfer (SET-PT), sequential proton loss electron transfer (SPLET) and finally radical adduct formation (RAF) [17,18,21]. The rationalization of results was performed on the basis of the O–H bond dissociation energies, electron transfer energies, adiabatic ionization potentials and proton affinities which are thermodynamic parameters related to the cited mechanisms. Kinetic aspects were taken into account evaluating the activation free energies ΔG for all involved reactions. 

## 2. Computational Details

The most common reaction mechanisms through which antioxidants exert their protection role and which we have examined can be divided roughly in two categories: one step (HAT and RAF) and two steps processes (SPLET and SET-PT) [17,18,21].

In the HAT reaction a hydrogen atom is transferred from the antioxidant to the free radical giving rise to another radical. In our particular case, highlighting in the formula of Vam3 only one of its hydroxyls as representative of all other ones, and considering the hydroperoxyl free radical ·OOH which we have chosen as counterpart, the HAT process is:

Vam3−OH + ⋅OOH → Vam3–O⋅ + HOOH(1)

The bond dissociation energy (BDE) referring to every single O–H bond of Vam3, represents the ability of Vam3−OH to transfer the hydrogen atom to ·OOH. The weaker the O–H bond, the easier the reaction of free radical inactivation will be. The way in which the BDE is calculated is the following:

BDE = G(Vam3–O⋅) + G(H⋅) − G(Vam3−OH)(2)

Although BDE is generally defined as the standard enthalpy change when a bond is cleaved by homolysis, we have computed this parameter and all others thermodynamic quantities in this work, in terms of free energy change in order to take into account the spontaneity of the reactions involved in the explored mechanisms.

The RAF process involves the addition of the free radical to the antioxidant carbon atoms:

Vam3−OH + ⋅OOH → [Vam3−OH]−OOH(3)

In this case, the most important factor is the presence of multiple bonds in the antioxidant. The process may be influenced by steric effects other than by the radical’s electrophilicity. The more electrophilic the radical, the more likely it will participates in RAF reactions. In the single electron transfer–proton transfer (SET-PT) we have two steps:

Transferring an electron to the free radical, Vam3−OH transforms in a radical cation. The radical cation in turn is deprotonated by the free radical anion giving rise to Vam3−O∙:

Vam3−OH + ⋅OOH → Vam3−OH⋅^+^ + ^−^OOH (4)

Vam3−OH⋅^+^ + ^−^OOH → Vam3−O⋅ + HOOH (5)

Adiabatic ionization potentials (IPs) and proton dissociation energy (PDE) provide information on energetics of these processes, respectively. The following equations are used for their evaluations:

IP = G(Vam3−OH⋅^+^) + G(e^−^) − G(Vam3−OH) (6)

PDE = G(Vam3−O⋅) + G(H^+^) − G(Vam3−OH⋅^+^) (7)

The SET-PT mechanisms is little common. There is evidences that it is involved in both the free radical scavenging processes and in the oxidation of biological targets [22].

The sequential proton loss electron transfer (SPLET) occurs through the steps in which Vam3−OH is transformed into an anion which in turn transfers an electron to a free radical:

Vam3−OH → Vam3−O^−^ + H⋅^+^ (8)

Vam3−O^−^ + ⋅OOH → Vam3−O⋅ + ^−^OOH (9)

Proton affinity (PA) and electron transfer energy (ETE) are the parameters which allow one to assess the efficiency of this mechanism:

PA = G(Vam3−O^−^) + G(H^+^) − G(Vam3−OH) (10)

ETE = G(Vam3−O⋅) + G(e^−^) − G(Vam3−O^−^) (11)

The SPLET mechanism was first proposed by Litwinienko and Ingold [23] mainly for the reactions of phenolic compounds. Although the three mechanisms involve a different number of steps, the final products are always the same. What changes, of course, are the energies at stake.

All calculations were performed using the hybrid B3LYP functional [24,25] as implemented in the Gaussian 09 software package [26]. B3LYP still represents the most widely used density functional, and its performance in determining some chemical properties, such as geometry, bond dissociation energies, binding energies and other thermochemical quantities, is usually accepted [27]. Furthermore its performance in describing antioxidant mechanisms is widely supported by literature papers [16,17,18,19,20]. Geometry optimizations of neutral, radical, radical cation and anion species of Vam3 were performed with the 6-311++G** basis set. The unrestricted open-shell approach was used for radical systems. Geometry optimizations, were followed by vibrational analysis of all species to evaluate their character as minima or saddle points by the number of imaginary frequencies: local minima present all real frequencies, while transition states are identified by the presence of a first-order saddle point (one imaginary frequency) corresponding to the expected motion along the reaction coordinate. In particular, to find the transition states, we have used flexible scans along the reaction pathway. After roughly knowing their geometry, an optimization was carried out to refine the structure. In order to ensure that the minima found corresponded to the same reaction coordinate as the transition state, the intrinsic reaction coordinate (IRC) calculations were performed [26].

Spin densities were obtained via the natural population analysis of Weinhold and Carpenter [28]. Solvation effects at 298.15 K were calculated with the Polarizable Continuum Model (PCM) [29] on the previously optimized geometries at the same level of theory, in water (ε = 80.0) and in an apolar (ε = 4.0) solvent. Dielectric constant ε = 4.0 is commonly used to describe a lipid-like environment [30] and was chosen hypothesizing a biochemical reaction environment to Vam3. To correct for thermal effects at the same temperature, free energy corrections were calculated at the same level of theory as the geometry optimizations. The solvation free energies for proton and electron were taken from a recent work [31].

## 3. Discussion

The chemical structure of an antioxidant is a key factor for its radical scavenging ability. As mentioned before, the concomitant presence of more than one hydroxyl groups, as well as the position of such groups on the rings and the planarity of some portion or of the entire molecule, is crucial for a good antioxidant activity. In particular, Vam3 contains five OH groups (see Figure 1). The homolytic or heterolytic breaking of the various O–H bonds present on Vam3 can generate as many radicals or anions which can be involved in the H or electron transfer reactions.

**Figure 2:**
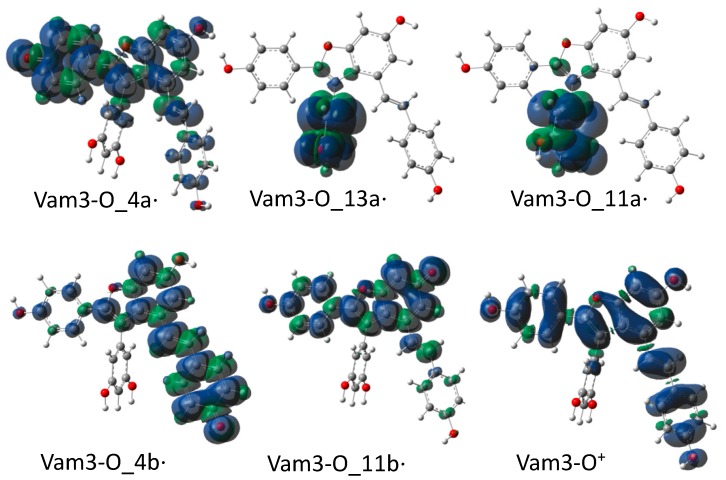
Spin density distribution in radical species of Vam3.

The stability of formed radicals is related to the way in which the unpaired electron is delocalized on the whole system. As it can be seen from Figure 2, the particular structure and disposition of conjugated bonds favor the delocalization in the Vam3–O_4a⋅, Vam3–O_4b⋅ and Vam3–O_11b⋅ radical species.

As far as Vam3–O_13a⋅ and Vam3–O_11b⋅ species are concerned, a perpendicular angle between rings B and E, prevents the electron density from spreading over the molecule and therefore this remains confined on the ring B on which the radicalizations took place. The most stable radical species is Vam3–O_4b⋅ in both media. Vam3–O_11b· and Vam3–O_4a⋅ lie at only 0.2 and 0.7 kcal/mol with respect to Vam3–O_4b⋅, irrespective of the solvent. The other two radicals are located at about 7–8 kcal/mol above the global minimum depending on the considered solvent.

Looking at the purely structural aspect, the arrangement of ring B with respect to the other rings of the molecule seems to have a role in determining the stability of the radicals. Probably the reasons can be ascribed to the effects of steric repulsion. In fact, as can be seen from the data shown in Figures S1 and S2 (see H–H distances), in the most stable radicals, this ring assumes an orientation that minimizes the repulsive interactions between its hydrogen atoms an those belonging to the adjacent rings A and D. 

Such a discussion can be also made for anions whose relative stability is Anion_4a > Anion_11b > Anion_4b > Anion_13a > Anion_11a. The first three species, are very similar from the energetic point of view since they are comprised in a narrow range of 1.4 kcal/mol. The other two lie at about 4 kcal/mol above the global minimum. The most important structural features for all anions can be found in Figures S3 and S4.

From Vam3_–_O^+^ radical cation spin distribution it can be observed that unpaired electron is well delocalized between A, E, C and D rings as with the more stable neutral radicals (see Figure 2). The values of thermodynamic parameters calculated and reported in Table 1 are consistent with the relative stability data (see BDEs).

The BDE indicates how hard it is to break the –OH bonds in an antioxidant. The values of this parameter show that the radicalization of 4b–OH, 11b–OH hydroxyls and 4a–OH of Vam3, requires almost the same amount of energy, in both solvents. However, the water facilitates the –OH cleavage. In fact, in this last medium we get lower values of 22–23 kcal/mol. Despite the absence of charged species in the radicalization processes, this relevant difference can be explained by the presence of many polar groups on the Vam3 that inevitably resents the polarity of the medium.

The similarity of the BDE values suggests that there is no significant difference in radical-scavenger activity between the three hydroxyl groups 4a–OH, 4b–OH and 11b–OH. For a more reliable comparison with the available literature data, our BDE value corresponding to the more stable radical of Vam3 (Vam3–O_4b⋅) was redone in terms of ΔH. We have obtained the value of 75.1 kcal/mol in the apolar solvent and 52.5 kcal/mol in water. 

In a previous study [16] on trans and cis resveratrol and some their derivatives, the computation performed at B3LYP/6-311++G(3df,2p) level in methanol indicated BDE values of 72.1 (*trans*-resveratrol) and 74.7 kcal/mol (*cis*-resveratrol). In two previous gas-phase B3LYP/6-311++G** investigations [17,32] the BDE of resveratrol was estimated to be 77.3 and 77.8 kcal/mol, respectively. Instead, the value of 76.9 kcal/mol was obtained using a gas-phase B3LYP/6-31G computational approach [33].

Taking into account the different basis set used in these investigations and that most of the data was obtained in the gaseous phase, all BDE available values are in any case slightly higher than those calculated for Vam3 in both solvents. Thus it is possible to hypothesize that Vam3 can act as a potent antioxidant with at least similar effectiveness than resveratrol and many other phenolic compounds [16,17,32,33].

The IP, adiabatic ionization potential, stands for the difficulty to extract an electron from neutral Vam3. Looking at the spin distribution of Vam3–O^+^· in Figure 2, it is quite likely that the stripped electron belonged to one of the three 4b–OH, 11b–OH or 4a–OH hydroxyls because only in these cases we can have a good delocalization.

As for the IP values in Table 1, there is a substantial difference between the values in the two media due to greater stabilization of a charged species in solvents with high polarity. 

The PDE, proton dissociation energy, is related to the difficulty for a hydroxyl radical cation of Vam3 to lose the proton with generating the corresponding radical. The PDE values in Table 1, say that the process concerning the loss of proton is more favorable for the usual three hydroxyls and slightly less easy in the apolar medium. Part of solvent effects difference derives from the proton free energy values, which are quite different in the two considered media [31]. 

As already mentioned, SET-PT is a stepwise mechanism. The energy required to accomplish the whole SET-PT reaction is equal to the sum of IP and PDE. The first step alone requires an energy equal to the IP that however, is already about 50 and 25 kcal/mol higher than the BDE values, depending on the considered solvent. This is sufficient to say that the HAT mechanism seems, at least from the thermodynamic point of view, favorable with respect to SET-PT. 

The difficulty for neutral Vam3 hydroxyl to lose the proton for generating the corresponding anion is measured by the PA values. 

As expected, the solvent effects are significant for PAs (see Table 1), since in this process are involved charged species, anion and proton. The different stabilization of anions and the difference of the values of G(H^+^) in the two considered solvents determines the big gap between the two set of PA values, thus, if we are in water, deprotonation of Vam3 appears to be a very low-cost energy process for all hydroxyl groups.

To extract an electron from a Vam3 anion in order to generate the corresponding radical an amount of energy equal to electron transfer free energy (ETE) is necessary. The values in Table 1 for this parameter, suggest the energetic requirement for radicalizing the anions increases in going from apolar to polar solvent. In water, the ETE values are comparable with IP. This means that extracting an electron from the neutral species or anions of Vam3 does not entail much difference. 

As SET-PT, SPLET is a stepwise mechanism. The amount of energy required for the whole process corresponds to the sum of PAs and ETEs. If we compare the amount of energy required for HAT and that individually required for each step of SPLET mechanisms, it is concluded that they could be competitive. More information on the mechanism used by the antioxidant Vam3 against the hydroperoxyl free radical, can be obtained evaluating the ΔG of the reactions involved in HAT, SET-PT and SPLET processes. In Table 2 we have reported the Gibbs free energies values (ΔG) for these reactions 

Data suggest that, in an apolar solvent, which better simulates the work environment of antioxidants, the HAT mechanism is the favored one because the first step of both SPLET and SET-PT is strongly endergonic. In water, despite the first two steps are in any case endergonic processes, the energies at stake are overall less prohibitive than those required in the apolar environment making all the three working mechanisms probable. 

More founded conclusions could be drawn by studying the entire course of the HAT process by evaluating the activation energies (ΔG^‡^) required to complete the transfer of the hydrogen atom from the antioxidant to the ·OOH free radical.

**Figure 3:**
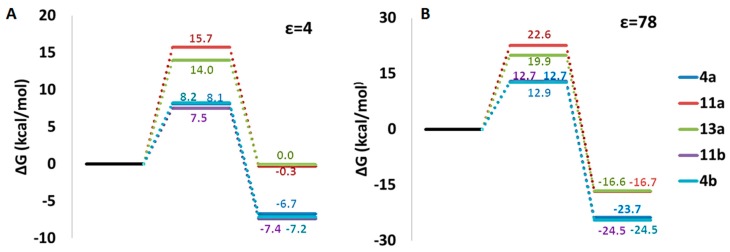
B3LYP/6-311++G** potential energy profiles in an apolar (**A**) and in water (**B**) solvents for HAT process from Vam3 to ·OOH free radical.

In Figure 3 we report the potential energy profiles obtained in the apolar and in water for the reaction of Vam3 with ·OOH. Computations indicate that, in order to transfer hydrogen atom from 4a−OH, 4b−OH or 11b−OH to free radical, we have to spend 7.5–8.2 kcal/mol in the non-polar solvent and 12.7–12.9 kcal/mol in water. This means the process is quite easy and this is a further support to the hypothesis that the HAT mechanism is favored over the others, especially in an apolar medium. Given the structural similarity of Vam3 with resveratrol, the present result is also supported by a previous DFT investigation [20] in which is found that the ·OOH scavenging activity of trans-resveratrol occurs by HAT. Moreover, in a previous theoretical work [16], it was found that some resveratrol derivatives act through the HAT mechanism because of the higher energies involved to form radical cations which prevents to follow the single electron transfer (SET) pathway. 

**Figure 4:**
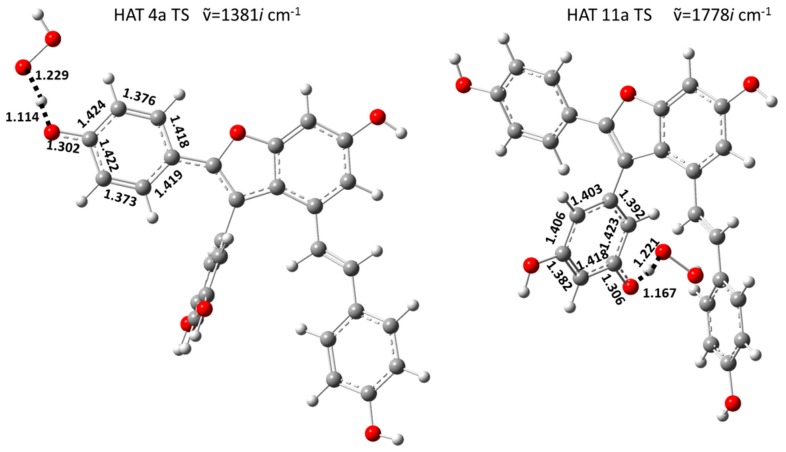

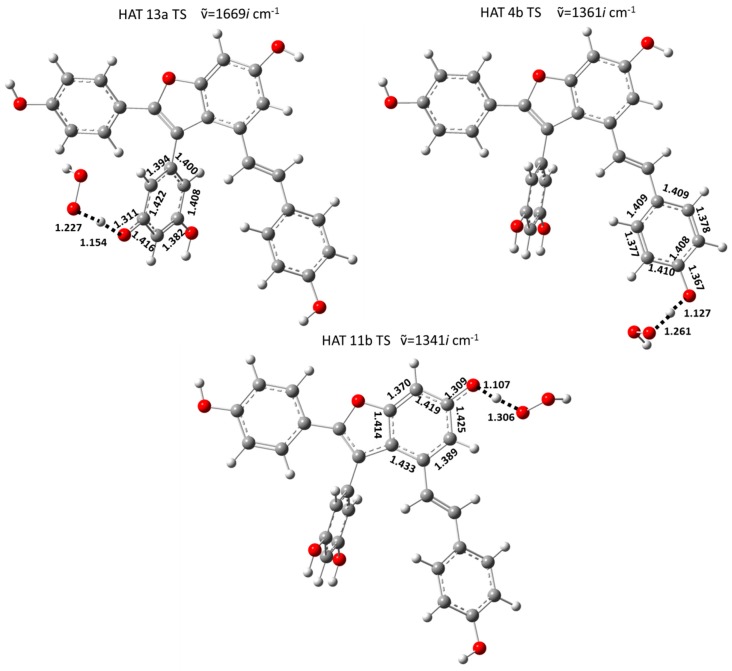
B3LYP/6-311++G** optimized structures of transition states intercepted studying HAT mechanism from Vam3 to ·OOH free radical. Imaginary frequencies (cm^−1^) and most significant geometrical parameters (Å) are also reported.

In Figure 4, we report optimized geometries of transition states lying on the PESs of Figure 3. In a RAF mechanism ·OOH free radical can bind to the carbon atoms of Vam3 to form the so called radical adducts. In Table 3 we have collected the reaction Gibbs free energy values obtained considering all the attack positions for ·OOH free radical on Vam3. In the apolar solvent the reaction appears to be endergonic in all cases except for the adduct formed on the C 11a (see Figure 1).

In water, we have found eight possible adducts formed mainly with the carbon atoms of the rings C and D where there is a good conjugate system and that is the part shared by Vam3 with resveratrol. Again, as has been done for the HAT mechanism, the activation energies (ΔG^‡^) for all processes providing negative ΔG values have been calculated and reported in Table 4. As can be observed, although the formation of the adducts examined is thermodynamically feasible, the reaction barriers are quite high and such as to prevent it from actually happening.

In an experimental work on the antioxidant properties of *trans*-resveratrol against superoxide anion and hydroxyl free radicals [34], it was observed the formation of two main radical adducts which then forms another one. In the first adduct, hydroxyl radical add in *ortho* position to the hydroxyl group of the phenol moiety because of its π-donor effect. In the second one, the hydroxyl adds to the aliphatic double bond generating a carbon-centred radical which is stabilized by resonance effects extending to the whole phenol moiety.

As can be noted (see Table 3), also our computations indicate that it is thermodynamically possible to obtain radical adducts on the two analogous attack positions on Vam3 (12b and 7b/8b), but the corresponding activation barriers (see Table 4) are very high, which makes us exclude the RAF mechanism. Since in Vam3 the attack positions for the formation of the radical adducts lying in the portion of resveratrol are exposed externally and that in any case the presence of the ring E does not imply problems for the effects of resonance and conjugation, the reason for this apparent disagreement can be ascribed to the different reactivity of the hydroperoxyl compared to the hydroxyl radical. In fact, as it occurs in the work above cited [34], adducts are formed with the hydroxyl and not with the less reactive superoxide anion radical. Moreover, this explanation is supported also by the results of a systematic study of the reactivity of *trans*-resveratrol toward hydroxyl and hydroperoxyl radicals in aqueous simulated media performed at density functional level [20] in which authors establish that ·OH scavenging activity of *trans*-resveratrol takes place predominantly by a RAF mechanism while the ·OOH scavenging activity of *trans*-resveratrol takes place almost exclusively by HAT.

## 4. Conclusions

A density functional-based investigation of the HAT, SET-PT, SPLET and RAF antioxidative mechanisms of Vam3, a natural product isolated from *Vitis Amurensis* Rupr. structurally similar to resveratrol, was performed, by examining its chemical behavior towards ·OOH free radical chosen as target in an apolar and a polar (water) solvent. Our results indicated that:

- Atom transfer seems to be the most probable mechanism followed by Vam3 in its action of radical scavenger especially in an apolar solvent due to the fact that the first step of both the SPLET and SET-PT pathways appears to be strongly endergonic. The height of activation barriers computed for the hydrogen atom transfer to the ·OOH free radical are quite low, and reinforce this hypothesis. In water, all three working mechanisms are probable because, although the first two steps of SET-PT and SPLET are in any case endergonic processes, the energies at stake are less prohibitive than those required in a lipid-like environment.
- Radical adduct formation, in rare cases favored thermodynamically, presents very high activation barriers that allow to permanently exclude this mechanism among those that Vam3 could adopt to counteract the action of free radicals.
- Based on the BDE value, Vam3 seems to be a good candidate useful for minimizing or preventing lipid oxidation, retarding the formation of toxic oxidation products even more effective than resveratrol and many other phenolic compounds.

The results of the present investigation are in good agreement with those obtained from the literature data concerning resveratrol and its derivatives.

## Figures and Tables

**Table 1:** BDE, IP, PDE, PA and ETE values (in kcal/mol) of Vam3 derived species for HAT, SET-PT and SPLET mechanisms in an apolar solvent (ε = 4) and in water (ε = 80).

Species	BDE		IP		PA		PDE		ETE	
	ε = 4.0	ε = 80.0	ε = 4.0	ε = 80.0	ε = 4.0	ε = 80.0	ε = 4.0	ε = 80.0	ε = 4.0	ε = 80.0
Vam3–O_4a·	68.1	45.7					16.0	12.6	55.7	65.9
Vam3–O_11a·	74.4	52.7					22.3	19.6	69.7	78.2
Vam3–O_13a·	74.7	52.9					22.6	19.8	68.3	76.7
Vam3–O_11b·	67.3	45.2					15.2	12.1	63.1	72.1
Vam3–O_4b·	67.6	45.0					15.5	11.9	61.3	72.3
Anion_4a					60.1	18.4				
Anion_11a					52.4	13.0				
Anion_13a					54.1	14.7				
Anion_11b					51.9	11.6				
Anion_4b					54.0	11.2				
Vam3^+^			117.6	71.6						

**Table 2:** Gibbs free energies values (ΔG) for all reactions between Vam3 most stable derived species with ·OOH free radical target involved in HAT, SET-PT and SPLET mechanisms.

	ΔG (kcal/mol)									
	4a		11a		13a		11b		4b	
	ε = 4.0	ε = 80.0	ε = 4.0	ε = 80.0	ε = 4.0	ε = 80.0	ε = 4.0	ε = 80.0	ε = 4.0	ε = 80.0
HAT	−6.7	−23.8	−0.3	−16.8	−0.0	−16.6	−7.4	−24.3	−7.2	−24.5
SPLET										
1° Step	60.1	18.4	52.4	13.0	54.2	14.7	52.9	11.6	54.0	11.2
2° Step	−66.8	−42.2	−52.7	−29.7	−54.2	−31.3	−59.3	−35.9	−61.1	−35.7
ΔΔG (kcal/mol)	−6.7	−23.8	−0.3	−16.7	−0.0	−16.6	−7.4	−24.3	−7.2	−24.5
SET-PT										
1° Step	44.4	4.6	44.4	4.6	44.4	4.6	44.4	4.6	44.4	4.6
2° Step	−51.1	−28.4	−44.7	−21.4	−44.5	−21.2	−51.8	−28.9	−51.6	−29.1
ΔΔG (kcal/mol)	−6.7	−23.8	−0.3	−16.8	−0.0	−16.6	−7.4	−24.3	−7.2	−24.5

**Table 3:** Gibbs free energy values for radical adduct formations between Vam3 and ·OOH free radical at 298.15 K.

ΔG (kcal/mol)			ΔG (kcal/mol)		
Species	ε = 4.0	ε = 80.0	Species	ε = 4.0	ε = 80.0
RAF_1a	31.9	37.5	RAF_8b	10.1	−11.3
RAF_2a	20.8	26.4	RAF_7b	6.4	−14.7
RAF_3a	23.8	50.1	RAF_1b	30.8	10.4
RAF_4a	18.2	24.1	RAF_2b	20.6	−1.4
RAF_5a	24.6	30.4	RAF_3b	24.4	1.8
RAF_6a	22.2	3.1	RAF_4b	16.2	−4.3
RAF_7a	14.5	−5.8	RAF_5b	24.9	10.4
RAF_8a	17.8	44.5	RAF_6b	20.7	1.5
RAF_14b	33.8	61.1	RAF_9a	36.6	14.8
RAF_13b	25.3	2.9	RAF_10a	23.1	2.2
RAF_12b	15.1	−7.7	RAF_11a	−5.2	55.4
RAF_11b	19.9	−1.8	RAF_12a	21.6	4.4
RAF_10b	18.0	−0.3	RAF_13a	24.9	4.5
RAF_9b	23.5	2.5	RAF_14a	23.4	1.6

**Table 4:** Gibbs free activation energy values for exergonic radical adduct formations between Vam3 and **·**OOH free radical at 298.15 K.

ΔG^‡^ (kcal/mol)		
Species	ε = 4.0	ε = 80.0
RAF_7a		26.3
RAF_12b		26.7
RAF_11b		30.3
RAF_10b		29.0
RAF_8b		24.4
RAF_7b		25.1
RAF_2b		31.4
RAF_4b		29.3
RAF_11a	29.1	

## Supplementary Materials

The following are available online. Figures S1 and S2: Optimized geometries of radical species of Vam3 and relative geometrical parameters. Figures S3 and S4: Optimized geometries of anion species of Vam3 and relative geometrical parameters. Figures S5 and S6: Spin densities of adducts obtained by exergonic radical adduct mechanism. Figures S7 and S8: Optimized structures and geometrical parameters of transition states encountered exploring RAF mechanism. Cartesian coordinates of all optimized species.

## References

[1] Pham-Huy L.A., He H., Pham-Huy C. (2008). Free radicals, antioxidants in disease and health. Int. J. Biomed. Sci..

[2] Lobo V., Patil A., Phatak A., Chandra N. (2010). Free radicals, antioxidants and functional foods: Impact on human health. Pharm. Rev..

[3] Alkadi H. (2018). A review on free radicals and antioxidants. Infect. Disord.-Drug Targets.

[4] Valko M., Leibfritz D., Moncol J., ronin M.T., Mazur M., Telser J. (2007). Free radicals and antioxidants in normal physiological functions and human disease. Int. J. Biochem. Cell Biol..

[5] Lin D., Xiao M., Zhao J., Li Z., Xing B., Li X., Kong M., Li L., Zhang Q., Liu Y. (2016). An overview of plant phenolic compounds and their importance in human nutrition and management of type 2 diabetes. Molecules.

[6] Burns J., Yokota T., Ashihara H., Lean M.E.J., Crozier A. (2002). Plant Foods and Herbal Sources of Resveratrol. J. Agric. Food Chem..

[7] De Lorgeril M., Salen P., Paillard F., Laporte F., Boucher F., de Leiris J. (2002). Mediterranean diet and the French paradox: Two distinct biogeographic concepts for one consolidated scientific theory on the role of nutrition in coronary heart disease. Cardiovasc. Res..

[8] Bradamante S., Barenghi L., Villa A. (2004). Cardiovascular Protective Effects of Resveratrol. Cardiovasc. Drug Rev..

[9] Parodi P.W. (1977). The French paradox unmasked: The role of folate. Med. Hypotheses.

[10] Kolouchova I., Melzoch K., Smidrkal J., Filip V. (2005). The content of resveratrol in vegetables and fruit. Chem. Listy.

[11] Gusman J., Malonne H., Atassi G. (2001). A reappraisal of the potential chemopreventive and chemotherapeutic properties of resveratrol. Carcinogenesis.

[12] De la Lastra C.A., Villegas I. (2005). Resveratrol as an anti-inflammatory and anti-aging agent: Mechanisms and clinical implications. Mol. Nutr. Food Res..

[13] Vastano B.C., Chen Y., Zhu N., Ho C.T., Zhou Z., Rosen R.T. (2000). Isolation and identification of stilbenes in two varieties of Polygonum cuspidatum. J. Agric. Food Chem..

[14] Shi J., Yin N., Xuan L.L., Yao C.S., Meng A.M., Hou Q. (2012). Vam3, a derivative of resveratrol, attenuates cigarette smoke-induced autophagy. Acta Pharmacol. Sin..

[15] Jiang M., Liu R., Chen Y., Zheng Q., Fan S., Liu P. (2014). A Combined Experimental and Computational Study of Vam3, a Derivative of Resveratrol, and Syk Interaction. Int. J. Mol. Sci..

[16] Mazzone G., Malaj N., Russo N., Toscano M. (2013). Density functional study of the antioxidant activity of some recently synthesized resveratrol analogues. Food Chem..

[17] Leopoldini M., Marino T., Russo N., Toscano M. (2004). Antioxidant properties of phenolic compounds: H-atom versus electron transfer mechanism. J. Phys. Chem. A.

[18] Leopoldini M., Russo N., Toscano M. (2011). The molecular basis of working mechanism of natural polyphenolic antioxidants. Food Chem..

[19] Leopoldini M., Chiodo S.G., Russo N., Toscano M. (2011). Detailed investigation of the OH radical quenching by natural antioxidant caffeic acid studied by quantum mechanical models. J. Chem. Theory Comput..

[20] Iuga C., Alvarez-Idaboy J.R., Russo N. (2012). Antioxidant activity of trans resveratrol toward hydroxyl and hydroperoxyl radicals: A quantum chemical and computational kinetics study. J. Org. Chem..

[21] Galano A., Mazzone G., Alvarez-Diduk R., Marino T., Alvarez-Idaboy J.R., Russo N. (2016). Food Antioxidants: Chemical Insights at the Molecular Level. Ann. Rev. Food Sci. Tech..

[22] Galano A., Alvarez-Idaboy J.R. (2009). Guanosine + OH radical reaction in aqueous solution: A reinterpretation of the UV-vis data based on thermodynamic and kinetic calculations. Org. Lett..

[23] Litwinienko G., Ingold K.U. (2003). Abnormal solvent effects on hydrogen atom abstractions. The reactions of phenols with 2,2-diphenyl-1-picrylhydrazyl (dpph·) in alcohols. J. Org. Chem..

[24] Becke A.D. (1993). Density-Functional Thermochemistry. III. The Role of Exact Exchange. J. Chem. Phys..

[25] Lee C., Yang W., Parr R.G. (1988). Development of the Colle-Salvetti Correlation-Energy Formula into a Functional of the Electron Density. Phys. Rev. B Condens. Matter Mater. Phys..

[26] Frisch M.J., Trucks G.W., Schlegel H.B., Scuseria G.E., Robb M.A., Cheeseman J.R., Scalmani G., Barone V., Mennucci B., Petersson G.A. (2009). Gaussian 09.

[27] Sousa S.F., Fernandes P.A., Ramos M.J. (2007). General Performance of Density Functionals. J. Phys. Chem. A.

[28] Weinhold F., Carpenter J., Naaman R., Vager Z. (1988). The Structure of Small Molecules and Ions.

[29] Vincenzo B., Cossi M., Tomasi J. (1997). A new definition of cavities for the computation of solvation free energies by the polarizable continuum model. J. Chem. Phys..

[30] Li L., Li C., Zhang Z., Alexov E. (2013). On the Dielectric “Constant” of Proteins: Smooth Dielectric Function for Macromolecular Modeling and Its Implementation in DelPhi. Chem. Theory Comput..

[31] Markovic Z., Tošovic J., Milenkovic D., Markovic S. (2016). Revisiting the solvation enthalpies and free energies of the proton and electron in various solvents. Comput. Theor. Chem..

[32] Nazarparvar E., Zahedi M., Klein E. (2012). Density Functional Theory (B3LYP) Study of Substituent Effects on O−H Bond Dissociation Enthalpies of trans-Resveratrol Derivatives and the Role of Intramolecular Hydrogen Bonds. J. Org. Chem..

[33] Lu L., Zhu S., Zhang H., Lia F., Zhanga S. (2015). Theoretical study of complexation of resveratrol with cyclodextrins and cucurbiturils: Structure and antioxidative activity. RSC Adv..

[34] Camont L., Collin F., Couturier M., Thérond P., Jore D., Gardès-Albert M., Bonnefont-Rousselot D. (2012). Radical-induced oxidation of *trans*-resveratrol. Biochimie.

