# Supplementary material for: Antioxidant Properties of the Vam3 Derivative of Resveratrol
Source: Molecules. 2018 Sep 25;23(10):2446. doi: 10.3390/molecules23102446 (PMC6222371; doi:10.3390/molecules23102446)
Supplement: Supplementary file 1 [file molecules-23-02446-s001.pdf]

Article

Antioxidant Properties of the Vam3 Derivative of Resveratrol

Seyedmohammad Ahmadi [1], Tiziana Marino [2], Mario Prejanò [2], Nino Russo [2] and Marirosa Toscano [2,*]

[1] Department of Food Science and Technology, Faculty of Agriculture, University of Zabol, P.O. Box 98615-538, Zabo 98613-35856 l, Iran; sma_257@yahoo.com

[2] Dipartimento di Chimica e Tecnologie Chimiche, Università della Calabria, Cubo 14C, Via P. Bucci, 87036 Arcavacata di Rende, CS, Italy; tiziana.marino65@unical.it (T.M.); mario.prejano@unical.it (M.P.); nrusso@unical.it (N.R.)

* Correspondence: m.toscano@unical.it; Tel.: +39-0984-492108

Academic Editor: Susana M. Cardoso

Received: 16 July 2018; Accepted: 20 September 2018; Published: 25 September 2018

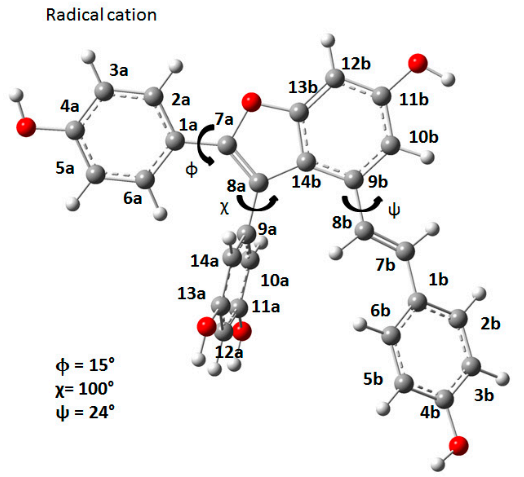

Bond type	Distance (Å)	Bond type	Distance (Å)
1a-2a	1.405	1b-2b	1.407
2a-3a	1.390	2b-3b	1.388
3a-4a	1.395	3b-4b	1.394
4a-5a	1.396	4b-5b	1.398
5a-6a	1.387	5b-6b	1.389
6a-1a	1.407	6b-1b	1.405
1a-7a	1.460	1b-7b	1.463
7a-8a	1.372	7b-8b	1.347
8a-9a	1.486	8b-9b	1.464
9a-10a	1.398	9b-10b	1.404
10a-11a	1.393	10b-11b	1.400
11a-12a	1.397	11b-12b	1.393
12a-13a	1.396	12b-13b	1.383
13a-14a	1.394	13b-14b	1.405
14a-9a	1.398	14b-9b	1.417
8a-14b	1.452		

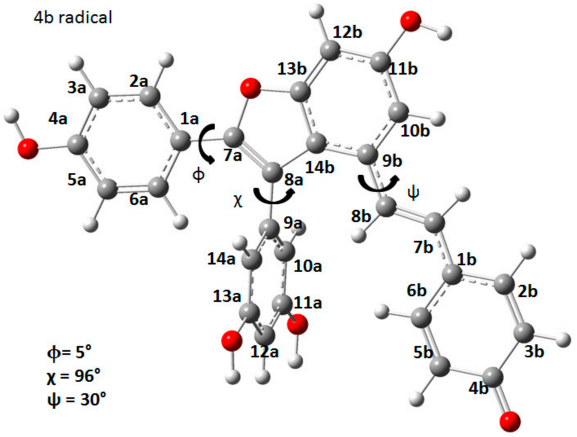

Bond type	Distance (Å)	Bond type	Distance (Å)	Bond type	Distance (Å)
1a-2a	1.406	1b-2b	1.431	H6a-H14a	3.360
2a-3a	1.389	2b-3b	1.364	H8b-H14a	3.333
3a-4a	1.396	3b-4b	1.456	H6a-H10a	3.256
4a-5a	1.396	4b-5b	1.461	H8b-H10a	3.218
5a-6a	1.386	5b-6b	1.361		
6a-1a	1.408	6b-1b	1.434		
1a-7a	1.458	1b-7b	1.426		
7a-8a	1.376	7b-8b	1.370		
8a-9a	1.487	8b-9b	1.445		
9a-10a	1.398	9b-10b	1.412		
10a-11a	1.394	10b-11b	1.393		
11a-12a	1.397	11b-12b	1.399		
12a-13a	1.397	12b-13b	1.380		
13a-14a	1.395	13b-14b	1.407		
14a-9a	1.398	14b-9b	1.424		
8a-14b	1.448				

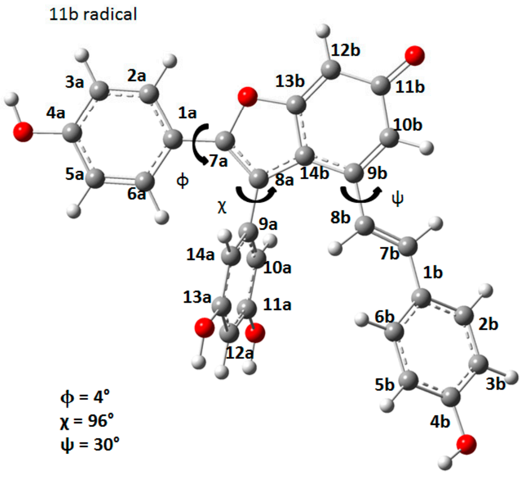

Bond type	Distance (Å)	Bond type	Distance (Å)	Bond type	Distance (Å)
1a-2a	1.409	1b-2b	1.406	H6a-H14a	3.253
2a-3a	1.387	2b-3b	1.389	H8b-H14a	3.230
3a-4a	1.397	3b-4b	1.394	H6a-H10a	3.350
4a-5a	1.397	4b-5b	1.398	H8b-H10a	3.542
5a-6a	1.384	5b-6b	1.389		
6a-1a	1.410	6b-1b	1.405		
1a-7a	1.451	1b-7b	1.464		
7a-8a	1.393	7b-8b	1.346		
8a-9a	1.487	8b-9b	1.468		
9a-10a	1.397	9b-10b	1.377		
10a-11a	1.394	10b-11b	1.461		
11a-12a	1.397	11b-12b	1.454		
12a-13a	1.396	12b-13b	1.361		
13a-14a	1.394	13b-14b	1.421		
14a-9a	1.398	14b-9b	1.442		
8a-14b	1.423				

Figure S1. Optimized geometries of radical species of Vam3 and relative geometrical parameters.

4a radical

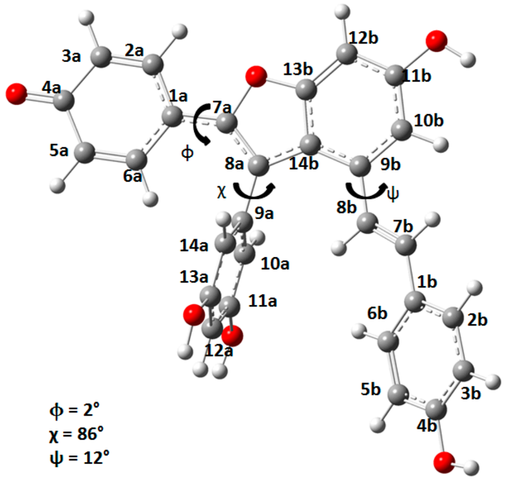

Bond type	Distance (Å)	Bond type	Distance (Å)	Bond type	Distance (Å)
1a-2a	1.431	1b-2b	1.404	H6a-H14a	3.334
2a-3a	1.364	2b-3b	1.391	H8b-H14a	3.488
3a-4a	1.457	3b-4b	1.394	H6a-H10a	3.228
4a-5a	1.457	4b-5b	1.396	H8b-H10a	3.194
5a-6a	1.364	5b-6b	1.384		
6a-1a	1.430	6b-1b	1.409		
1a-7a	1.429	1b-7b	1.461		
7a-8a	1.393	7b-8b	1.348		
8a-9a	1.486	8b-9b	1.461		
9a-10a	1.398	9b-10b	1.401		
10a-11a	1.394	10b-11b	1.402		
11a-12a	1.397	11b-12b	1.396		
12a-13a	1.397	12b-13b	1.379		
13a-14a	1.394	13b-14b	1.411		
14a-9a	1.397	14b-9b	1.424		
8a-14b	1.437				

13a radical

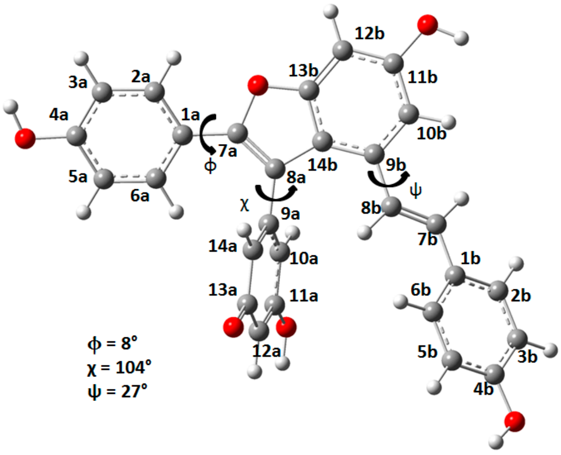

Bond type	Distance (Å)	Bond type	Distance (Å)	Bond type	Distance (Å)
1a-2a	1.405	1b-2b	1.406	H6a-H14a	3.401
2a-3a	1.390	2b-3b	1.388	H8b-H14a	3.495
3a-4a	1.397	3b-4b	1.395	H6a-H10a	3.379
4a-5a	1.396	4b-5b	1.398	H8b-H10a	3.308
5a-6a	1.386	5b-6b	1.388		
6a-1a	1.407	6b-1b	1.405		
1a-7a	1.460	1b-7b	1.463		
7a-8a	1.372	7b-8b	1.347		
8a-9a	1.484	8b-9b	1.465		
9a-10a	1.410	9b-10b	1.403		
10a-11a	1.417	10b-11b	1.401		
11a-12a	1.372	11b-12b	1.393		
12a-13a	1.449	12b-13b	1.373		
13a-14a	1.455	13b-14b	1.405		
14a-9a	1.383	14b-9b	1.417		
8a-14b	1.452				

11a radical

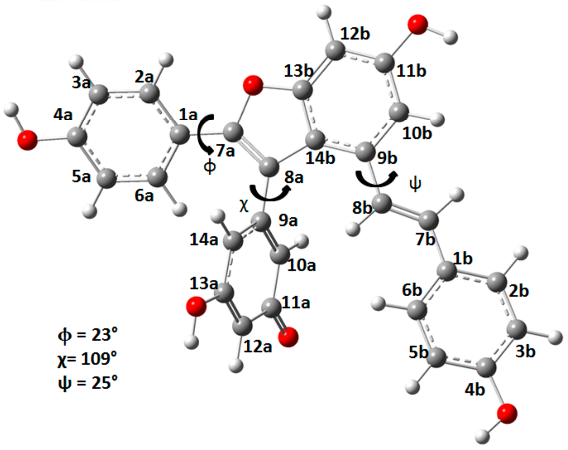

Bond type	Distance (Å)	Bond type	Distance (Å)	Bond type	Distance (Å)
1a-2a	1.404	1b-2b	1.407	H6a-H14a	3.428
2a-3a	1.389	2b-3b	1.388	H8b-H14a	3.750
3a-4a	1.396	3b-4b	1.394	H6a-H10a	3.339
4a-5a	1.396	4b-5b	1.398	H8b-H10a	3.062
5a-6a	1.387	5b-6b	1.387		
6a-1a	1.406	6b-1b	1.405		
1a-7a	1.460	1b-7b	1.462		
7a-8a	1.372	7b-8b	1.347		
8a-9a	1.484	8b-9b	1.465		
9a-10a	1.385	9b-10b	1.403		
10a-11a	1.454	10b-11b	1.400		
11a-12a	1.449	11b-12b	1.392		
12a-13a	1.372	12b-13b	1.382		
13a-14a	1.417	13b-14b	1.406		
14a-9a	1.408	14b-9b	1.418		
8a-14b	1.454				

Figure S2. Optimized geometries of radical species of Vam3 and relative geometrical parameters.

4a anion

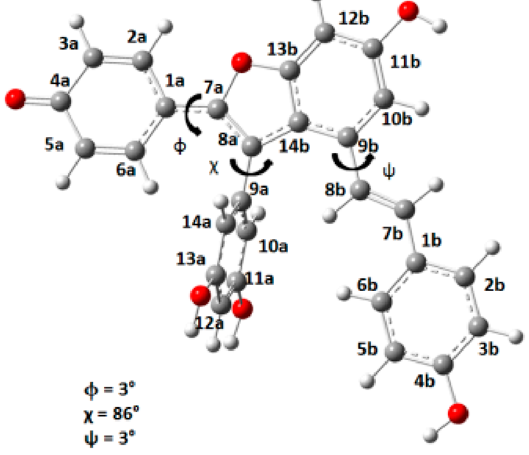

Bond type	Distance (Å)	Bond type	Distance (Å)
1a-2a	1.425	1b-2b	1.409
2a-3a	1.372	2b-3b	1.390
3a-4a	1.452	3b-4b	1.392
4a-5a	1.452	4b-5b	1.396
5a-6a	1.372	5b-6b	1.389
6a-1a	1.424	6b-1b	1.408
1a-7a	1.429	1b-7b	1.461
7a-8a	1.394	7b-8b	1.353
8a-9a	1.486	8b-9b	1.454
9a-10a	1.400	9b-10b	1.418
10a-11a	1.391	10b-11b	1.386
11a-12a	1.397	11b-12b	1.402
12a-13a	1.397	12b-13b	1.376
13a-14a	1.391	13b-14b	1.416
14a-9a	1.400	14b-9b	1.421
8a-14b	1.440		

13a anion

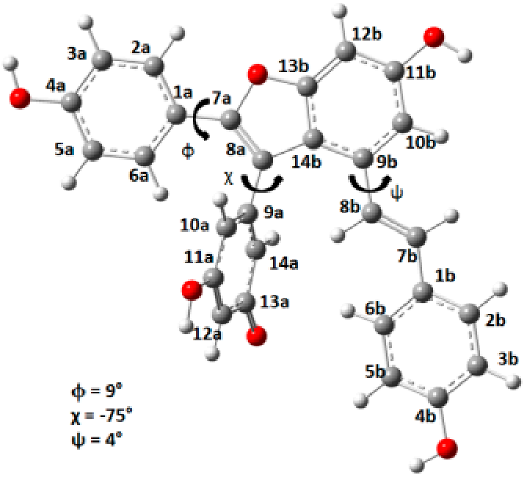

Bond type	Distance (Å)	Bond type	Distance (Å)
1a-2a	1.404	1b-2b	1.406
2a-3a	1.392	2b-3b	1.391
3a-4a	1.393	3b-4b	1.393
4a-5a	1.395	4b-5b	1.396
5a-6a	1.387	5b-6b	1.387
6a-1a	1.409	6b-1b	1.408
1a-7a	1.459	1b-7b	1.466
7a-8a	1.370	7b-8b	1.348
8a-9a	1.489	8b-9b	1.461
9a-10a	1.405	9b-10b	1.406
10a-11a	1.399	10b-11b	1.396
11a-12a	1.383	11b-12b	1.393
12a-13a	1.443	12b-13b	1.385
13a-14a	1.447	13b-14b	1.407
14a-9a	1.392	14b-9b	1.420
8a-14b	1.456		

11b anion

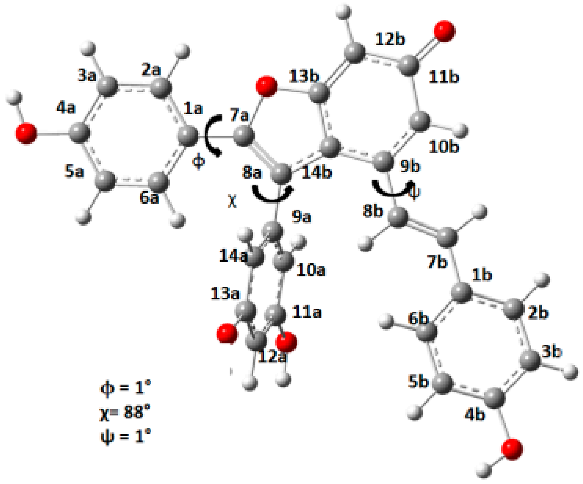

Bond type	Distance (Å)	Bond type	Distance (Å)
1a-2a	1.409	1b-2b	1.406
2a-3a	1.387	2b-3b	1.388
3a-4a	1.397	3b-4b	1.394
4a-5a	1.397	4b-5b	1.398
5a-6a	1.384	5b-6b	1.389
6a-1a	1.410	6b-1b	1.405
1a-7a	1.451	1b-7b	1.464
7a-8a	1.394	7b-8b	1.346
8a-9a	1.487	8b-9b	1.468
9a-10a	1.397	9b-10b	1.377
10a-11a	1.394	10b-11b	1.461
11a-12a	1.397	11b-12b	1.454
12a-13a	1.396	12b-13b	1.361
13a-14a	1.394	13b-14b	1.421
14a-9a	1.398	14b-9b	1.442
8a-14b	1.423		

Figure S3. Optimized geometries of anion species of Vam3 and relative geometrical parameters.

11a anion

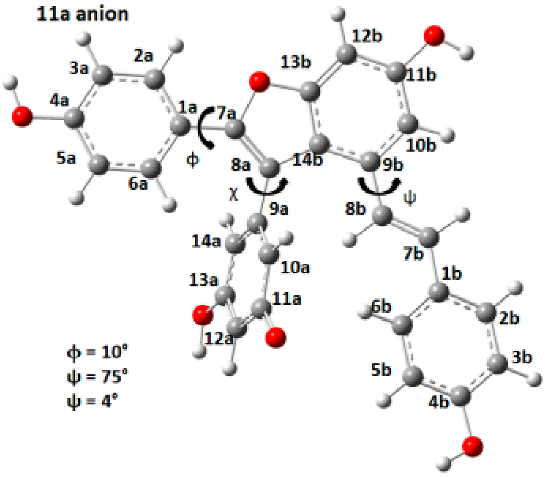

Bond type	Distance (Å)	Bond type	Distance (Å)
1a-2a	1.404	1b-2b	1.406
2a-3a	1.392	2b-3b	1.391
3a-4a	1.393	3b-4b	1.393
4a-5a	1.395	4b-5b	1.396
5a-6a	1.387	5b-6b	1.387
6a-1a	1.409	6b-1b	1.408
1a-7a	1.459	1b-7b	1.466
7a-8a	1.370	7b-8b	1.348
8a-9a	1.489	8b-9b	1.461
9a-10a	1.391	9b-10b	1.406
10a-11a	1.447	10b-11b	1.396
11a-12a	1.443	11b-12b	1.393
12a-13a	1.383	12b-13b	1.385
13a-14a	1.399	13b-14b	1.407
14a-9a	1.405	14b-9b	1.420
8a-14b	1.456		

4b anion

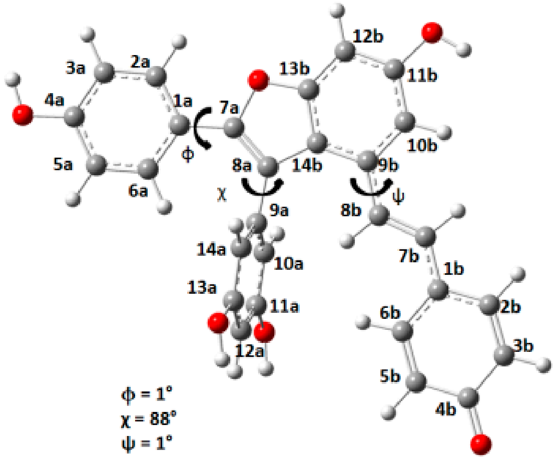

Bond type	Distance (Å)	Bond type	Distance (Å)
1a-2a	1.407	1b-2b	1.424
2a-3a	1.391	2b-3b	1.373
3a-4a	1.393	3b-4b	1.451
4a-5a	1.394	4b-5b	1.456
5a-6a	1.388	5b-6b	1.370
6a-1a	1.409	6b-1b	1.425
1a-7a	1.457	1b-7b	1.427
7a-8a	1.373	7b-8b	1.371
8a-9a	1.487	8b-9b	1.441
9a-10a	1.398	9b-10b	1.406
10a-11a	1.392	10b-11b	1.396
11a-12a	1.396	11b-12b	1.396
12a-13a	1.396	12b-13b	1.386
13a-14a	1.392	13b-14b	1.404
14a-9a	1.398	14b-9b	1.433
8a-14b	1.451		

Figure S4. Optimized geometries of anion species of Vam3 and relative geometrical parameters.

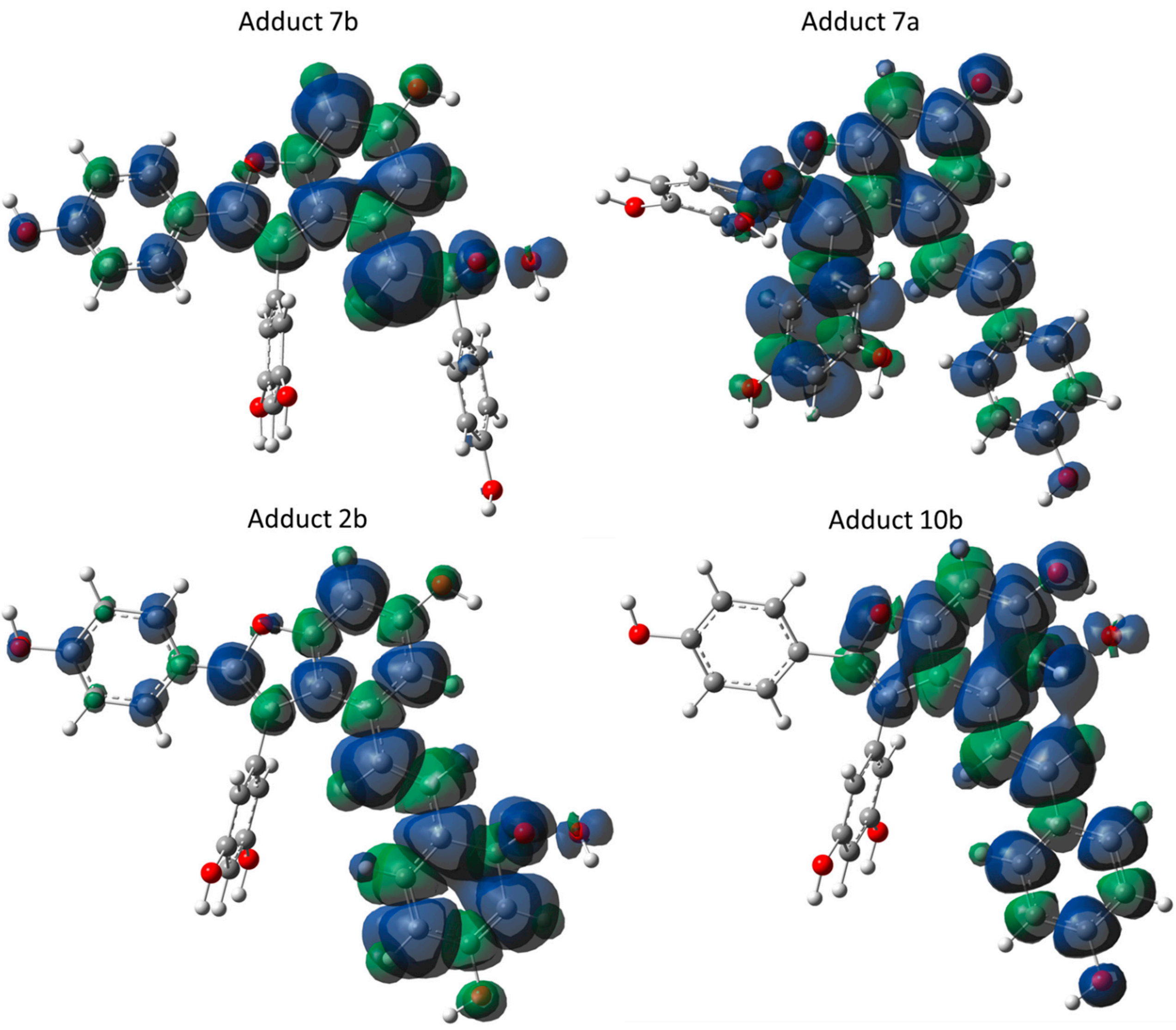

Figure S5. Spin densities of adducts obtained by exergonic radical adduct formation mechanism.

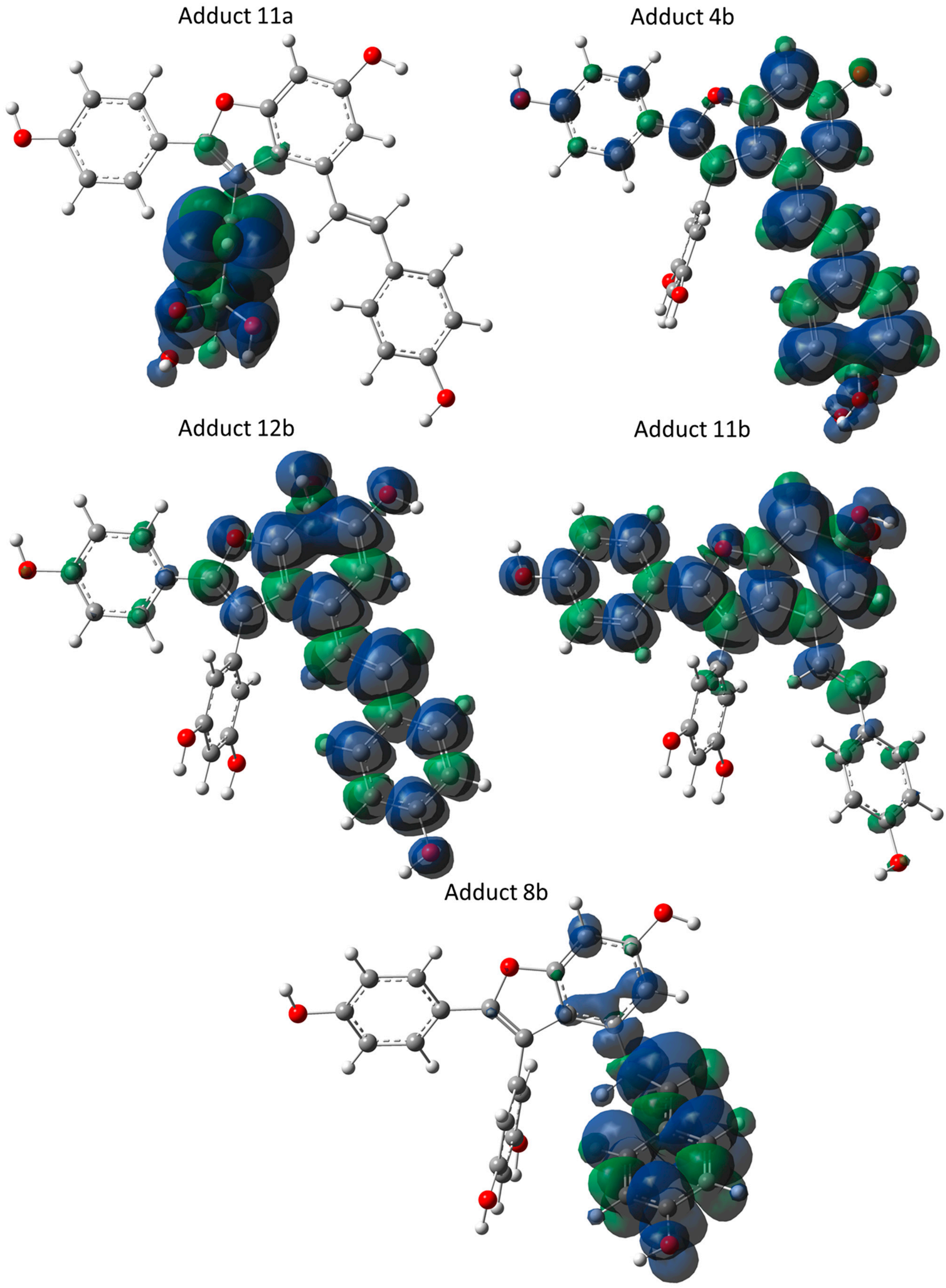

Figure S6. Spin densities of adducts obtained by exergonic radical adduct formation mechanism.

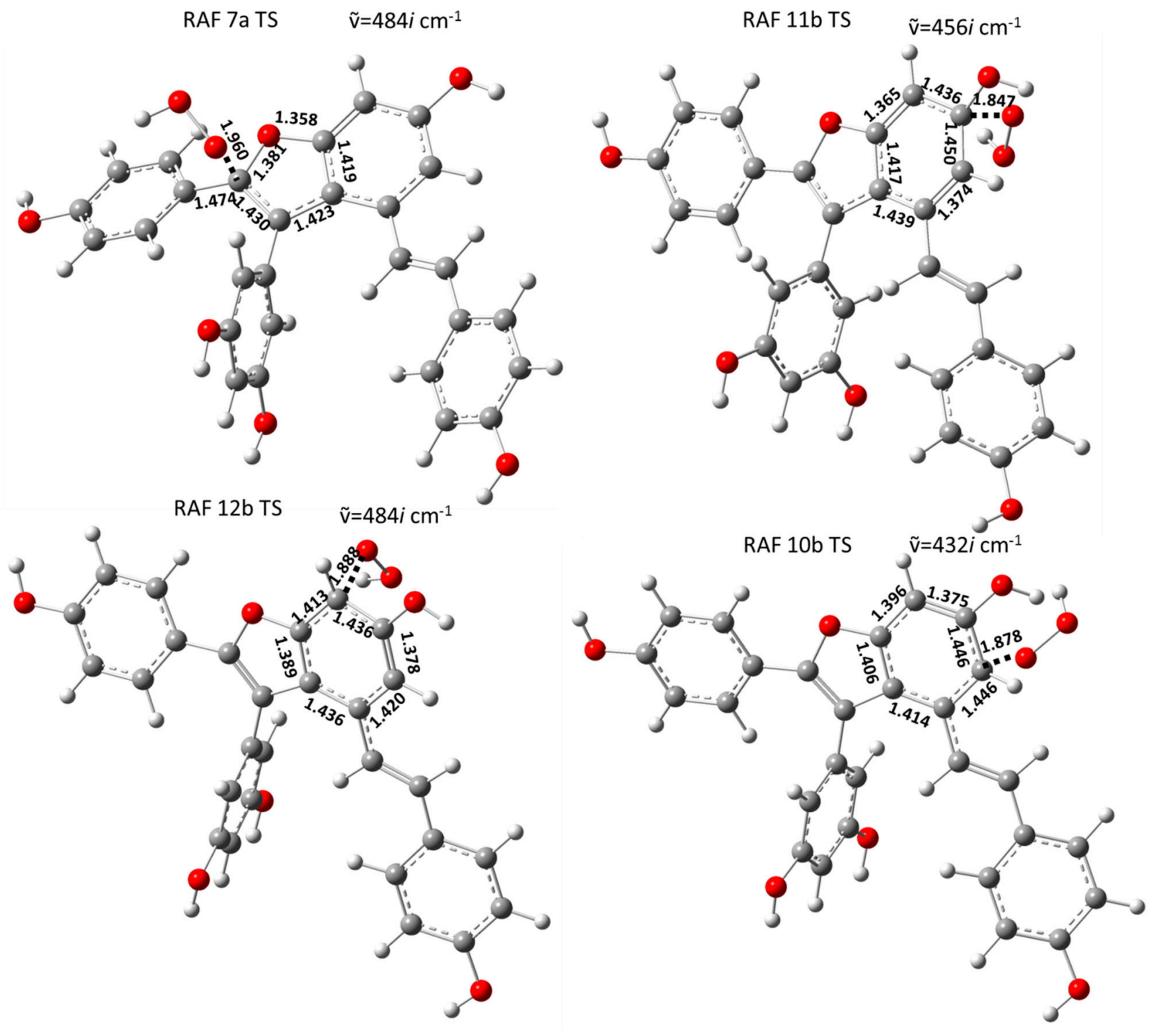

Figure S5. Optimized geometries of RAF Transition State species of Vam3 and relative geometrical parameters.

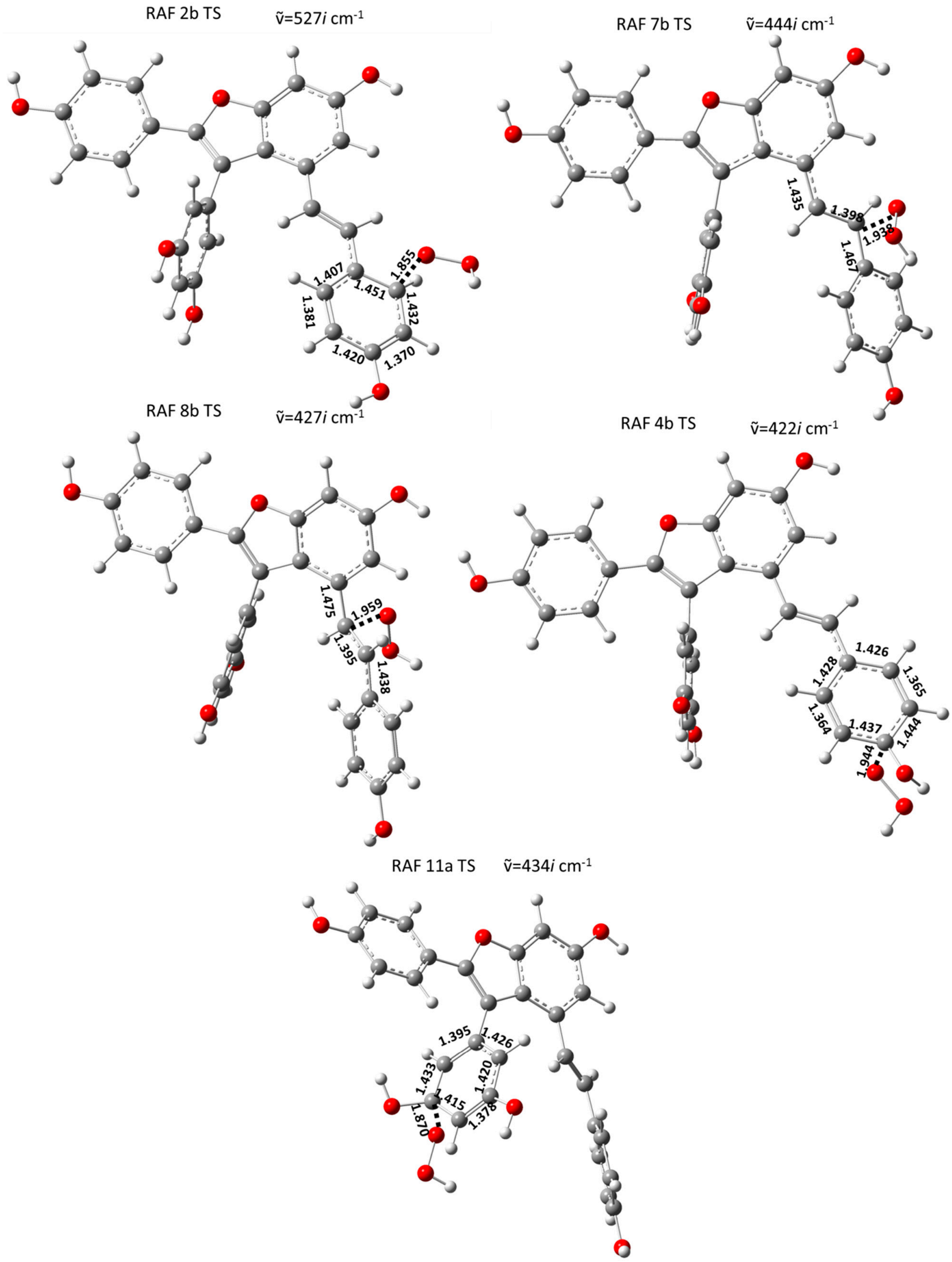

Figure S6. Optimized geometries of RAF Transition State species of Vam3 and relative geometrical parameters.

Cartesian coordinates 4a HAT TS

C	0.90937200	2.82240100	0.08728100
C	-0.08071600	1.81789800	0.11529000
C	-1.44859700	2.20995700	0.15174100
C	-1.70530900	3.58807000	0.16921500
C	-0.68423400	4.54641100	0.13465400
C	0.66238900	4.17923400	0.09217100
C	1.98700700	0.88929800	0.03973000
C	0.63644800	0.57090200	0.07977700
H	-2.73105300	3.93261300	0.24797900
H	1.44969300	4.92005900	0.07407700
C	3.20745600	0.12935200	-0.01186000
C	4.45655300	0.80163600	-0.01050900
C	3.22148100	-1.28887900	-0.07108800
C	5.64763900	0.11459800	-0.06092500
H	4.46961900	1.88246600	0.03242700
C	4.40438400	-1.98429800	-0.12397000
H	2.29056900	-1.83654500	-0.07813600
C	5.65551700	-1.30774400	-0.11921000
H	6.59179600	0.64491000	-0.05795100
H	4.41571200	-3.06644900	-0.17135400
C	0.04545800	-0.79275200	0.08182200
C	-0.13739600	-1.47179400	1.29041300
C	-0.34053300	-1.38079600	-1.12511900
C	-0.70214800	-2.74672600	1.28168900
H	0.15957700	-1.02963000	2.23255100
C	-0.90477500	-2.65585400	-1.11837900
H	-0.20786600	-0.86532400	-2.06724800
C	-1.08796300	-3.34727300	0.08131200
H	-1.52499000	-4.34265200	0.08042100
C	-2.52671800	1.22465900	0.18488300
H	-2.24142500	0.23218700	0.50934000
C	-3.80736200	1.44875300	-0.17406800
H	-4.07320900	2.42457200	-0.57363600
C	-4.91419500	0.49651600	-0.12294500
C	-4.81879500	-0.77834200	0.46975100
C	-6.15085700	0.85405900	-0.68363200
C	-5.89487500	-1.64914200	0.49011400
H	-3.88965000	-1.09228500	0.93004000
C	-7.23936600	-0.01158100	-0.66963200
H	-6.26150800	1.83034500	-1.14410300
C	-7.11350800	-1.27028400	-0.08254200
H	-5.81650600	-2.62556400	0.95292400
H	-8.18291900	0.29238800	-1.11319100
O	6.74662300	-2.01717800	-0.16764100
O	-1.25991200	-3.18582100	-2.32530200
O	-0.85703200	-3.36821500	2.48879200
O	-0.95396200	5.88522600	0.15878300
O	-8.13884900	-2.17230300	-0.02955000
O	2.14624100	2.26480000	0.04639100
H	-1.90591200	6.02721500	0.19099600
H	-8.92503800	-1.80076600	-0.44361900
H	-1.64996000	-4.05799900	-2.20560000
H	-1.22607600	-4.24902400	2.36541900
H	7.72623100	-1.48726900	-0.18085300
O	8.95065000	-1.05340400	-0.21636300
O	8.87496900	0.34815600	-0.15653800
H	9.80494300	0.60697000	-0.22484200

Cartesian coordinates 11b HAT TS

C	1.31651300	2.60537400	-0.12855200
C	0.51540600	1.43970600	-0.14344700
C	-0.90828100	1.58835600	-0.21331700
C	-1.40162400	2.88588500	-0.27227000
C	-0.56683200	4.03944700	-0.22604200
C	0.84271500	3.89040900	-0.15235300
C	2.70617300	0.88832100	-0.03075200
C	1.43125700	0.34082900	-0.07587900
H	-2.46422900	3.05671300	-0.38833200
H	1.48512300	4.75982200	-0.12207600
C	4.04948100	0.34434800	0.05259400
C	5.15664100	1.21323100	-0.00643400
C	4.30420200	-1.03472300	0.20008600
C	6.45401900	0.72951900	0.07231700
H	4.99267100	2.27661100	-0.11594200
C	5.59801600	-1.52061700	0.28065800
H	3.48292800	-1.73376500	0.25712500
C	6.68219700	-0.64144300	0.21571800
H	7.29155500	1.41896600	0.02251600
H	5.78773100	-2.58062000	0.39623200
C	1.09129500	-1.10626900	-0.04791300
C	0.75554300	-1.71343600	1.16460800
C	1.09864900	-1.84858600	-1.23265200
C	0.43056400	-3.06904000	1.18719700
H	0.73891600	-1.14957000	2.08801000
C	0.77367300	-3.20383200	-1.19450800
H	1.35611400	-1.39133500	-2.17921800
C	0.43964100	-3.82322900	0.01162400
H	0.18906300	-4.88079500	0.03576700
C	-1.79448600	0.42464900	-0.25190400
H	-1.34961000	-0.49981600	-0.59924700
C	-3.08768400	0.42847600	0.12878500
H	-3.49777500	1.33998800	0.55634000
C	-4.02642600	-0.68993100	0.06372500
C	-3.76758500	-1.88183100	-0.63498500
C	-5.26719000	-0.58194500	0.71845700
C	-4.69380000	-2.91545600	-0.66526600
H	-2.83686000	-2.00109500	-1.17682200
C	-6.20053900	-1.60863700	0.69731800
H	-5.49976500	0.33029400	1.25773200
C	-5.91504000	-2.78403200	0.00325300
H	-4.47599300	-3.82335200	-1.22097700
H	-7.15212700	-1.51377100	1.20598700
O	7.93268500	-1.17780100	0.29902400
O	0.79843900	-3.88532700	-2.37956900
O	0.11450800	-3.61370900	2.39860200
O	-1.06207800	5.24987500	-0.28287900
O	-6.86541100	-3.76785000	0.00295800
O	2.63639500	2.26205700	-0.06668300
H	8.59536200	-0.48048100	0.24700700
H	-0.13612300	-4.53750200	2.29464100
H	-2.12704700	5.29847700	0.01578100
H	0.57333000	-4.81038100	-2.23587200
H	-6.56618800	-4.51098700	-0.53120400
O	-3.31904400	5.36190000	0.54491400
O	-3.97763100	6.35762600	-0.19178100
H	-4.54896800	6.76518400	0.47547700

Cartesian coordinates 13a HAT TS

C	1.07214900	3.13820300	-0.10297400
C	0.16142500	2.06883200	-0.05862400
C	-1.21908600	2.34658400	-0.21874400
C	-1.57803700	3.69291500	-0.38029000
C	-0.63443600	4.72834600	-0.38783800
C	0.72807400	4.46906300	-0.25661300
C	2.27778700	1.30827600	0.18738600
C	0.97470800	0.88257500	0.13837400
H	-2.62020600	3.94086600	-0.55389600
H	1.46021800	5.26429900	-0.28649300
C	3.56544800	0.64003500	0.35529500
C	4.73755600	1.24499000	-0.12689500
C	3.68558300	-0.60291200	1.00065600
C	5.97469700	0.62611200	0.00697800
H	4.67546000	2.20774600	-0.61749300
C	4.91803400	-1.22865200	1.13590700
H	2.80946200	-1.08147600	1.41669300
C	6.07059900	-0.61695800	0.63509600
H	6.86615700	1.11160000	-0.37895800
H	5.00765700	-2.17701800	1.65268400
C	0.51763900	-0.52372500	0.25320700
C	-0.19405300	-0.93857100	1.38499500
C	0.79815900	-1.44272000	-0.75719400
C	-0.61071000	-2.27771000	1.51153400
H	-0.43726600	-0.24088400	2.17646000
C	0.41610500	-2.80513600	-0.61477400
H	1.31942400	-1.13778700	-1.65329400
C	-0.30179600	-3.20843400	0.53718900
H	-0.58406200	-4.25165800	0.62861900
C	-2.22128500	1.27885800	-0.25730600
H	-1.85558300	0.30715500	-0.56745900
C	-3.52260500	1.40927800	0.06501900
H	-3.86664900	2.36238600	0.46053500
C	-4.55450400	0.37500900	-0.02031400
C	-4.37951800	-0.83633700	-0.71105200
C	-5.79513000	0.58483800	0.60810900
C	-5.38349500	-1.79426200	-0.75512500
H	-3.45264800	-1.03211100	-1.23690800
C	-6.80693600	-0.36472900	0.57213000
H	-5.96506100	1.51373600	1.14286100
C	-6.60240600	-1.56365100	-0.11043900
H	-5.22527700	-2.71892600	-1.30290900
H	-7.75727700	-0.19232300	1.06254300
O	7.25522400	-1.27749400	0.79799200
O	0.70020400	-3.69840900	-1.53118500
O	-1.30034500	-2.58712400	2.64631100
O	-1.00231800	6.03768200	-0.55361200
O	-7.62787400	-2.46859600	-0.12441000
O	2.34729600	2.68142700	0.03535300
H	-1.95970800	6.09790500	-0.63482600
H	-7.37492700	-3.24142300	-0.64027500
H	-1.55178400	-3.51723200	2.63613300
H	7.97699300	-0.75385300	0.43382500
H	1.77359600	-3.67094800	-1.95387600
O	2.98626500	-3.73912300	-2.12706800
O	3.40326900	-2.42723400	-1.93375300
H	4.10391900	-2.49980500	-1.26315800

Cartesian coordinates 11a HAT TS

C	1.66798200	2.91260100	0.31559300
C	0.65396900	1.94517200	0.18366800
C	-0.69905300	2.37845600	0.23254100
C	-0.91713600	3.74666800	0.46537600
C	0.12824700	4.66407300	0.60577000
C	1.46204200	4.26172900	0.52454500
C	2.69534300	0.98770400	-0.02385100
C	1.35073600	0.69383100	-0.04586900
H	-1.93589400	4.10532100	0.56820700
H	2.27339200	4.96923900	0.62667600
C	3.90869100	0.19764200	-0.17395600
C	5.11761700	0.83019900	-0.51412300
C	3.92733200	-1.19574800	0.02979600
C	6.28942000	0.10200000	-0.66419100
H	5.13332700	1.90162900	-0.66518100
C	5.09549600	-1.92670100	-0.11808400
H	3.02470800	-1.71033600	0.32785600
C	6.28270700	-1.28188200	-0.47042600
H	7.21120000	0.60956300	-0.93287500
H	5.10697400	-2.99691000	0.04765300
C	0.76859400	-0.64612700	-0.30653500
C	0.09780600	-1.34682900	0.69149200
C	0.85870400	-1.18824700	-1.59782600
C	-0.49116500	-2.61146500	0.41072100
H	0.01381600	-0.94390400	1.69348000
C	0.27214600	-2.43378400	-1.88290100
H	1.36439200	-0.65278300	-2.39089200
C	-0.38588400	-3.14668600	-0.89865100
H	-0.83316300	-4.11537700	-1.09420400
C	-1.81068000	1.44977000	0.05215400
H	-1.57935000	0.40503900	0.21285600
C	-3.06596100	1.77935600	-0.31959100
H	-3.28138200	2.81364900	-0.57763100
C	-4.20018900	0.87017400	-0.45762600
C	-4.18872500	-0.45815500	0.00604900
C	-5.37860600	1.32804600	-1.07632100
C	-5.28849600	-1.28746100	-0.15684600
H	-3.31764000	-0.85175200	0.51529500
C	-6.48525800	0.50851500	-1.24481600
H	-5.42149300	2.35046800	-1.43761000
C	-6.44228900	-0.80846800	-0.78575600
H	-5.25453100	-2.30700100	0.21606600
H	-7.38595300	0.87087400	-1.72515700
O	7.40232900	-2.05108300	-0.60347000
O	0.40195700	-2.88267500	-3.16539800
O	-1.14035200	-3.28885700	1.31949000
O	-0.10110300	5.99357000	0.83737700
O	-7.55546000	-1.57952200	-0.96840100
O	2.89518700	2.33592100	0.19167800
H	-1.04897100	6.15725600	0.87998400
H	-7.40808100	-2.45794400	-0.60183800
H	8.15642500	-1.49744600	-0.83276500
H	-0.03446200	-3.73641500	-3.25941100
O	-0.40003800	-2.84329200	3.54034800
O	-1.52093000	-2.99108100	4.36952700
H	-1.13587500	-3.38866700	5.16445700
H	-0.84049900	-3.01466400	2.41381200

Cartesian coordinates 4b HAT TS

C	-2.63301112	2.78884232	-0.10757714
C	-1.44563412	2.03912832	-0.03908714
C	-0.21000512	2.73107132	0.03440086
C	-0.27104312	4.13411732	0.02831886
C	-1.47860812	4.83516632	-0.05539014
C	-2.70150812	4.16967532	-0.12365414
C	-3.23914412	0.66164032	-0.13221314
C	-1.86947412	0.65026132	-0.05932314
H	0.64821188	4.70203332	0.12783586
H	-3.63701412	4.70967632	-0.17482214
C	-4.27350512	-0.36707668	-0.17882314
C	-5.60994812	-0.02814868	0.09017786
C	-3.98763312	-1.70719768	-0.49762214
C	-6.61578612	-0.98646268	0.05613186
H	-5.86071312	0.99643132	0.33110186
C	-4.98782812	-2.66701968	-0.53331114
H	-2.97424212	-2.00245368	-0.73109714
C	-6.30833212	-2.31183668	-0.25334714
H	-7.64129912	-0.70078368	0.27203186
H	-4.76175412	-3.69632968	-0.78387914
C	-1.00119212	-0.55342868	0.01647586
C	-0.31905912	-0.99733668	-1.12113314
C	-0.85773312	-1.23094368	1.23078886
C	0.50105888	-2.12056268	-1.03758614
H	-0.41446912	-0.48010168	-2.06696114
C	-0.03554512	-2.35460168	1.29960886
H	-1.37712012	-0.89838168	2.11996586
C	0.64867688	-2.80768668	0.16988986
H	1.28665088	-3.68617068	0.22836386
C	1.06146288	2.01421232	0.13254286
H	0.98566188	0.98201532	0.44964086
C	2.28020388	2.51602632	-0.14992614
H	2.35157988	3.53412032	-0.52536814
C	3.56407388	1.82553532	-0.03721914
C	3.70451288	0.51815232	0.46260686
C	4.73663988	2.48460832	-0.44891114
C	4.94530788	-0.09702668	0.54137986
H	2.83260788	-0.03134468	0.79676786
C	5.98459588	1.88092932	-0.37604914
H	4.66381388	3.49528532	-0.83768114
C	6.09500088	0.58247732	0.12235386
H	5.02951988	-1.10804768	0.92747386
H	6.87950188	2.40067932	-0.69651314
O	-7.25343912	-3.29985368	-0.30221214
O	0.06766088	-2.98156168	2.51091986
O	1.13966788	-2.51656868	-2.18006814
O	-1.51931012	6.20619932	-0.05378614
O	7.34122688	0.02868632	0.18489486
O	-3.71254612	1.96307732	-0.15982414
H	-8.11945912	-2.93221868	-0.09686514
H	1.70512888	-3.27400768	-1.99733914
H	-0.62356712	6.55499032	-0.00289514
H	0.66253688	-3.73552668	2.44343986
H	7.27743600	-1.03146500	0.56190800
O	7.27821120	-2.14334304	0.37790905
O	6.84915220	-2.63813604	-0.77609695
H	7.63375620	-2.67117604	-1.35854895

Cartesian coordinates 4b radical

C	1.53527338	2.77794243	0.00895209
C	0.53282698	1.79056700	0.01324689
C	-0.82829522	2.20834292	0.00648401
C	-1.06466705	3.60059609	0.00471391
C	-0.03241687	4.53613542	0.00550821
C	1.30919033	4.13906728	0.00607601
C	2.58275434	0.83090618	0.01383375
C	1.24086297	0.52759924	0.01718397
H	-2.08539917	3.96606706	-0.00110281
H	2.11014931	4.86574353	0.00356295
C	3.81537537	0.05189588	0.01041580
C	5.05430843	0.70689190	-0.10259001
C	3.82386920	-1.35181027	0.12052224
C	6.24656541	-0.00515895	-0.11006516
H	5.08149438	1.78486194	-0.18655826
C	5.01121859	-2.06626941	0.11415200
H	2.89434131	-1.89354119	0.21693780
C	6.23097426	-1.39676811	-0.00227017
H	7.19061551	0.52409821	-0.20037402
H	5.01032851	-3.14592117	0.20065713
C	0.63936306	-0.83266887	0.01663786
C	0.29373094	-1.44152116	1.22710436
C	0.40143227	-1.48775432	-1.19498330
C	-0.28634098	-2.70974206	1.21867192
H	0.46996390	-0.94617210	2.17312935
C	-0.17888996	-2.75617419	-1.18917611
H	0.66114823	-1.02858987	-2.13989913
C	-0.52666419	-3.37500518	0.01386603
H	-0.97442117	-4.36560541	0.01291722
C	-1.90226732	1.24100214	-0.00345182
H	-1.58916796	0.20667600	-0.01478574
C	-3.24711045	1.50378998	-0.00032915
H	-3.57924114	2.53804045	0.01494026
C	-4.29850309	0.54025610	-0.01400679
C	-4.07469339	-0.87583333	-0.03738002
C	-5.65499922	0.99527392	-0.00412917
C	-5.11058855	-1.75821311	-0.04981304
H	-3.05850638	-1.25206195	-0.04531186
C	-6.70906262	0.13014215	-0.01600708
H	-5.83470253	2.06622231	0.01355805
C	-6.50220353	-1.31347085	-0.03959410
H	-4.94388555	-2.82933824	-0.06783894
H	-7.73486161	0.48008389	-0.00829009
O	7.36785655	-2.15309999	-0.00338515
O	-0.38405409	-3.35240032	-2.40015909
O	-0.59861113	-3.25992024	2.42865898
O	-0.26743824	5.88467961	0.00326714
O	-7.44888946	-2.11776418	-0.05034381
O	2.76582903	2.20209598	0.00807313
H	-1.21549527	6.05259126	0.00344600
H	8.14075273	-1.58483188	-0.08928120
H	-0.81091704	-4.20780846	-2.28433541
H	-1.01600286	-4.11936442	2.30828704

Cartesian coordinates 4a radical

C	1.68128714	2.73996496	0.06402304
C	0.66085084	1.76603911	0.10095591
C	-0.69452503	2.19916328	0.15312326
C	-0.90871905	3.58352642	0.17619327

C	0.14121095	4.51123340	0.13154076
C	1.47498527	4.10422730	0.07378001
C	2.70101434	0.77423130	0.00106894
C	1.33669591	0.49851725	0.05478890
H	-1.92267610	3.95816539	0.26827011
H	2.28409623	4.82089734	0.04845094
C	3.88789753	-0.01934990	-0.06374205
C	5.16611459	0.62308716	-0.08468000
C	3.85206112	-1.44761093	-0.11352598
C	6.32281245	-0.09767077	-0.14721923
H	5.20256114	1.70394704	-0.04884782
C	4.99999738	-2.18185918	-0.17801576
H	2.89818737	-1.95478817	-0.10214920
C	6.31426729	-1.55360990	-0.19792076
H	7.29118564	0.38916806	-0.16160015
H	4.97342528	-3.26473734	-0.21752413
C	0.70097888	-0.84472511	0.05840582
C	0.50138327	-1.51799733	1.26745201
C	0.29000395	-1.41743725	-1.14739016
C	-0.10519991	-2.77362646	1.25990012
H	0.81743227	-1.08733595	2.20871334
C	-0.31601724	-2.67334102	-1.13920114
H	0.43547001	-0.90583027	-2.08971725
C	-0.51594517	-3.35968550	0.06066118
H	-0.98553389	-4.34009430	0.06096916
C	-1.80335933	1.24812011	0.19763924
H	-1.55021734	0.25086918	0.53398694
C	-3.07561416	1.51029992	-0.16409628
H	-3.30847120	2.48958898	-0.57573321
C	-4.21416758	0.59647907	-0.10084690
C	-4.16117948	-0.67309124	0.50798793
C	-5.43853239	0.98862111	-0.66497313
C	-5.26628361	-1.50642793	0.54081915
H	-3.24270399	-1.01246425	0.97165833
C	-6.55597853	0.16083284	-0.63858729
H	-5.51652465	1.96207811	-1.13796234
C	-6.47215367	-1.09372894	-0.03541625
H	-5.22068335	-2.47876995	1.01642408
H	-7.48923341	0.49109501	-1.08508324
O	7.36409463	-2.21930800	-0.25512376
O	-0.69427208	-3.18929730	-2.34477116
O	-0.27484408	-3.39079636	2.46693808
O	-0.08868005	5.85680256	0.16079527
O	-7.52736063	-1.95963913	0.03042187
O	2.89988815	2.14740816	0.00903623
H	-1.03553108	6.02738549	0.20359089
H	-8.30107650	-1.56746217	-0.38809383
H	-1.11287087	-4.04810912	-2.22471301
H	-0.67075492	-4.26002926	2.34462934

Cartesian coordinates 11a radical

C	1.61229301	2.75408712	-0.04122185
C	0.60351025	1.77708410	-0.07399485
C	-0.74782006	2.19809328	-0.13254091
C	-0.97907316	3.58189920	-0.13290022
C	0.06049078	4.51889853	-0.07624227
C	1.39490223	4.12005023	-0.03565279
C	2.64328005	0.79565715	0.00156478
C	1.30296086	0.50540819	-0.04053286
H	-1.99816906	3.94268854	-0.22744091
H	2.19989037	4.84172312	-0.01220706
C	3.86826611	0.00316395	0.05420574
C	5.09688021	0.59455181	-0.28301828
C	3.87645936	-1.34655501	0.44978584
C	6.28000941	-0.13262822	-0.24110691
H	5.12281731	1.63368817	-0.58344385
C	5.05387133	-2.07724296	0.49335618
H	2.95517447	-1.83233232	0.73946594
C	6.26360439	-1.47377504	0.14465695
H	7.21723577	0.34566017	-0.51021630
H	5.05357023	-3.11526699	0.80256715
C	0.68472890	-0.84401231	-0.04605906
C	0.05825501	-1.32048986	1.12389098
C	0.69080703	-1.63942710	-1.17700821
C	-0.55447086	-2.59720194	1.15980995
H	0.03548874	-0.71319917	2.02042202
C	0.06678217	-2.95340226	-1.17901009
H	1.15500184	-1.31054185	-2.09857833
C	-0.55728820	-3.40277746	0.04855307
H	-1.01758986	-4.38510453	0.04496419
C	-1.85344331	1.24117889	-0.22694190
H	-1.60800403	0.28380993	-0.67150900
C	-3.11586444	1.45627303	0.19118909
H	-3.33728701	2.38317302	0.71561486
C	-4.25317959	0.54595428	0.05120795
C	-4.24528532	-0.57666614	-0.79415905
C	-5.42815523	0.79256729	0.78394699
C	-5.34528131	-1.41862525	-0.88732229
H	-3.37583123	-0.79054795	-1.40434223
C	-6.53462464	-0.04115094	0.70023485
H	-5.46960462	1.65641528	1.43942721
C	-6.49550256	-1.15577391	-0.13730509
H	-5.31570824	-2.27529442	-1.55463121
H	-7.43293583	0.15781812	1.27191932
O	7.39212250	-2.24141808	0.20603410
O	0.06455592	-3.66255110	-2.20978492
O	-1.11600783	-2.94183709	2.35357138
O	-0.18261695	5.86739033	-0.08553515
O	-7.60951275	-1.94705001	-0.19232788
O	2.83885920	2.16558804	-0.00459802
H	-1.13171328	6.02568154	-0.11607503
H	-7.46737944	-2.66944945	-0.81310519
H	-1.52536930	-3.81178120	2.28716440
H	8.15850663	-1.71893810	-0.05318178

Cartesian coordinates 13a radical

C	-1.49634128	2.80123522	0.04931297
C	-0.51599491	1.79360607	0.06851522
C	0.84583321	2.17812637	0.15859230
C	1.11069224	3.55537525	0.21040987
C	0.09866827	4.52160739	0.16793228
C	-1.24422007	4.16037248	0.09020197
C	-2.58531027	0.88088327	-0.06222701
C	-1.25642713	0.54473030	-0.00610618
H	2.13672724	3.88833727	0.32770889
H	-2.03045628	4.90253828	0.07385886
C	-3.82894348	0.12156313	-0.15441974
C	-5.03367528	0.69520608	0.28334584
C	-3.87587621	-1.17629594	-0.69406888

C	-6.23171141	-0.00397783	0.20428994
H	-5.02884337	1.69807846	0.69038528
C	-5.06852425	-1.87937512	-0.77470279
H	-2.97451855	-1.63584510	-1.07612586
C	-6.25355955	-1.29711186	-0.32141221
H	-7.15082085	0.45825109	0.55239596
H	-5.10020239	-2.87549996	-1.19882724
C	-0.71774321	-0.83801409	0.01634681
C	0.00061120	-1.35481388	-1.04907908
C	-0.94036226	-1.64872792	1.14632016
C	0.51709402	-2.71358495	-1.02415483
H	0.19698306	-0.76937080	-1.93885729
C	-0.45271325	-2.97842599	1.20433016
H	-1.49534325	-1.26686303	1.99426108
C	0.25837979	-3.50965645	0.15846583
H	0.64171314	-4.52444749	0.17221809
C	1.92802503	1.19227391	0.21740083
H	1.64706789	0.20914619	0.57444307
C	3.20709036	1.41315091	-0.14420292
H	3.46450810	2.38514897	-0.55841112
C	4.32692150	0.47505194	-0.07810021
C	4.22796852	-0.82456029	0.44764109
C	5.58314542	0.87684406	-0.56713513
C	5.32246318	-1.67674045	0.47691094
H	3.28353795	-1.18318741	0.83893486
C	6.68662445	0.03551996	-0.54176902
H	5.69295023	1.87436592	-0.98012517
C	6.55863023	-1.25059348	-0.01882178
H	5.21807239	-2.67868148	0.88322699
H	7.64778179	0.35680570	-0.92414086
O	-7.39859593	-2.03542102	-0.42446313
O	-0.74115613	-3.65581097	2.35317714
O	1.15528389	-3.17715427	-1.99531309
O	0.37790717	5.86193928	0.22514799
O	7.66959130	-2.04732328	-0.01390572
O	-2.74054018	2.25335526	-0.02395215
H	1.33060138	5.99279846	0.26989416
H	7.44871536	-2.91420663	0.34247717
H	-8.14784053	-1.52595192	-0.09798511
H	-0.38502513	-4.55010623	2.30623542

Cartesian coordinates 11b radical

C	-1.64816646	2.80943324	0.08379786
C	-0.62320411	1.82514503	0.12098897
C	0.75224292	2.25431625	0.18973809
C	0.97668499	3.61233218	0.21316688
C	-0.07569933	4.62324012	0.14801670
C	-1.45128342	4.15661638	0.08893670
C	-2.65324022	0.83825380	0.00967019
C	-1.28719297	0.56758229	0.06646202
H	1.98190109	4.00151241	0.31875097
H	-2.25816442	4.87640063	0.05322676
C	-3.85061646	0.02285622	-0.06516130
C	-5.11569564	0.63994781	-0.00397095
C	-3.80956448	-1.37961430	-0.20799788
C	-6.28228435	-0.10629001	-0.07452086
H	-5.17833753	1.71447608	0.10148297
C	-4.97250133	-2.12697398	-0.28096031
H	-2.85984425	-1.89038042	-0.26834790
C	-6.21716168	-1.49525699	-0.21278799
H	-7.24598544	0.39153084	-0.02254930
H	-4.93557164	-3.20352635	-0.39328083
C	-0.65550033	-0.77866527	0.05791369
C	-0.18646830	-1.31143087	-1.14481306
C	-0.52458187	-1.49763829	1.24984311
C	0.41141785	-2.57101719	-1.15137010
H	-0.27297026	-0.76184484	-2.07290212
C	0.07385198	-2.75664728	1.22772509
H	-0.88228188	-1.09660025	2.18937826
C	0.54205319	-3.30287038	0.03095581
H	1.00416516	-4.28683314	0.01980075
C	1.85003806	1.28250022	0.25916191
H	1.61548712	0.32950386	0.71881585
C	3.09122224	1.49626296	-0.21548733
H	3.28357023	2.42089119	-0.75467820
C	4.23800702	0.59200699	-0.11843199
C	4.27135947	-0.52777599	0.72941104
C	5.38129341	0.84578611	-0.89711895
C	5.38222317	-1.35924204	0.78322784
H	3.42595648	-0.74649923	1.37096331
C	6.49749756	0.02193016	-0.85383807
H	5.39016718	1.70909699	-1.55426396
C	6.50063929	-1.08891501	-0.01098307
H	5.38840766	-2.21332935	1.45486384
H	7.37160048	0.22739593	-1.45967141
O	-7.32678181	-2.28313842	-0.28787031
O	0.17734688	-3.41981908	2.41896128
O	0.84902680	-3.04635542	-2.35385819
O	0.19820969	5.84021761	0.15713918
O	7.62337878	-1.87072095	0.00496686
O	-2.87070614	2.19356405	0.02426278
H	-8.12142188	-1.74131912	-0.23207703
H	0.58664325	-4.28098732	2.28507732
H	1.29432098	-3.89201029	-2.23713016
H	7.51633151	-2.58269719	0.64428124

Cartesian coordinates RAF 11b

C	1.67078509	2.44923234	-0.29838612
C	0.64261327	1.46074405	-0.28527416
C	-0.73525686	1.89093023	-0.40693625
C	-0.96397785	3.22964125	-0.50387305
C	0.09012524	4.29541957	-0.44655998
C	1.48899312	3.78580019	-0.39349303
C	2.66839110	0.47493499	-0.10668425
C	1.29680706	0.21250912	-0.15719210
H	-1.97631933	3.59556732	-0.63996421
H	2.30019592	4.50022440	-0.43427090
C	3.85740739	-0.34043190	0.02577622
C	5.12882664	0.25617788	-0.09481586
C	3.80639735	-1.72651010	0.28640184
C	6.28926473	-0.49362606	0.03045521
H	5.20045343	1.31729098	-0.29152586
C	4.96338943	-2.47622038	0.41335411
H	2.85270618	-2.22119028	0.39873823
C	6.21328069	-1.86517835	0.28388084
H	7.25713256	-0.01106880	-0.06972810
H	4.91710653	-3.53900515	0.61714027
C	0.65331588	-1.12617915	-0.08779580
C	0.12604579	-1.57586286	1.12464612

C	0.56634719	-1.92020590	-1.23523729
C	-0.48683778	-2.82647221	1.18570524
H	0.17765592	-0.96703226	2.01762214
C	-0.04846523	-3.16871707	-1.15889130
H	0.96944796	-1.58391217	-2.18175811
C	-0.57540723	-3.63191227	0.04831812
H	-1.05060417	-4.60829721	0.10180418
C	-1.83668533	0.91677895	-0.45086378
H	-1.62691735	-0.02031723	-0.95507709
C	-3.04539126	1.11639095	0.10276199
H	-3.19649835	2.02004101	0.68899619
C	-4.19918816	0.21497720	0.04273583
C	-4.29781251	-0.85401800	-0.86289230
C	-5.27762547	0.41700808	0.92173310
C	-5.40753160	-1.68953701	-0.87577829
H	-3.50537329	-1.02937305	-1.58091439
C	-6.39199867	-0.41116120	0.92018791
H	-5.23537860	1.24086032	1.62654799
C	-6.45932959	-1.47271087	0.01871224
H	-5.46480763	-2.50414929	-1.59261397
H	-7.21561067	-0.24645107	1.60426327
O	7.31846685	-2.65561146	0.41708481
O	-0.10972014	-3.90732839	-2.30886701
O	-0.98170582	-3.22013405	2.39628127
O	-0.00695921	5.21545825	-1.51015386
O	-7.57841935	-2.26118444	0.04733914
O	2.89236007	1.83151209	-0.19901615
H	8.11573640	-2.12630192	0.30773721
H	-1.43601295	-4.06476709	2.31188809
H	-0.89328506	5.60037861	-1.49477703
H	-0.53480928	-4.75365056	-2.13583822
H	-7.51920759	-2.93487100	-0.63796710
O	-0.18356312	5.19446738	0.70727185
O	-0.25014103	4.42034245	1.92743923
H	0.64295089	4.54503936	2.28067510

Cartesian coordinates RAF 2b

C	1.73943790	2.78984521	0.02990486
C	0.84407317	1.70622197	0.02199896
C	-0.55290820	1.98009131	0.01687176
C	-0.92963213	3.33996306	0.03306511
C	0.00222889	4.37769552	0.04924206
C	1.37617095	4.12262045	0.04639509
C	2.98132429	0.96056730	0.02296682
C	1.67885134	0.52177088	0.02126287
H	-1.98238635	3.59723952	-0.00627287
H	2.09892123	4.92700850	0.04886899
C	4.28729935	0.31023122	0.02385002
C	5.44410569	1.06747698	-0.22828019
C	4.44705849	-1.06375634	0.28360990
C	6.70291570	0.47977485	-0.23005610
H	5.35368729	2.12721528	-0.42592418
C	5.70130435	-1.65438906	0.28397292
H	3.58431754	-1.67806604	0.49827806
C	6.83735371	-0.88564110	0.02495600
H	7.58145260	1.08610984	-0.43063810
H	5.81766355	-2.71146824	0.48974508
C	1.22745785	-0.89451487	0.01210916
C	0.83873324	-1.50808637	1.20731207
C	1.18843526	-1.60746003	-1.18955923
C	0.41820190	-2.83649113	1.19418901
H	0.86313413	-0.96951305	2.14572326
C	0.76770919	-2.93674746	-1.18813416
H	1.48751090	-1.14680595	-2.12197696
C	0.37941585	-3.56025003	-0.00032809
H	0.05490902	-4.59793433	-0.00378520
C	-1.53067006	0.91533694	-0.03117224
H	-1.14096595	-0.06719916	-0.26131725
C	-2.87918726	1.04889016	0.19559291
H	-3.25738919	2.02329704	0.48854279
C	-3.86659338	0.03572793	0.10093897
C	-3.61890721	-1.28758985	-0.31391112
C	-5.29034953	0.44380614	0.41315620
C	-4.61208612	-2.23763711	-0.35535099
H	-2.61561413	-1.57278516	-0.60758227
C	-6.28072328	-0.67145925	0.46323594
H	-5.32465133	1.03075314	1.33870625
C	-5.95197244	-1.91857022	0.06340178
H	-4.38898922	-3.24376234	-0.69784826
H	-7.28450954	-0.45612115	0.80661694
O	8.04729844	-1.52185291	0.03834418
O	0.75402307	-3.58912352	-2.38976816
O	0.05655317	-3.38947130	2.39074238
O	-0.37642812	5.69399959	0.05269600
O	-6.90993566	-2.90206413	0.06791514
O	3.02369128	2.34328629	0.02486498
H	8.75072688	-0.89058203	-0.14652124
H	-0.21931804	-4.30326039	2.26522630
H	-1.33733886	5.75591438	0.04295014
H	0.46907700	-4.50088322	-2.26963540
H	-6.50759955	-3.74920917	-0.14688479
O	-5.60992867	1.40855236	-0.65788427
O	-6.81541349	2.12591139	-0.26907023
H	-7.45578464	1.76158926	-0.89644901

Cartesian coordinates RAF 2b

C	-2.28605524	2.73720107	-0.07376995
C	-1.20501333	1.84008529	-0.01775178
C	0.11799912	2.37035900	0.00032015
C	0.23185584	3.77873726	-0.03285291
C	-0.87634927	4.62209656	-0.08413389
C	-2.17856294	4.11369534	-0.10733196
C	-3.16701206	0.70929013	-0.03601792
C	-1.80527390	0.52184709	0.00841498
H	1.21717911	4.23114729	-0.03550091
H	-3.03841617	4.76795157	-0.15145582
C	-4.33118719	-0.16978705	-0.04105886
C	-5.61543052	0.37557720	-0.21379078
C	-4.23017731	-1.56385424	0.12786616
C	-6.74573037	-0.43169804	-0.22336519
H	-5.72745947	1.44364792	-0.34237821
C	-5.35550448	-2.37319645	0.11950886
H	-3.26356204	-2.02381193	0.27302900
C	-6.62124672	-1.81174203	-0.05754485
H	-7.72629052	0.01483284	-0.36056391
H	-5.26957827	-3.44478514	0.25205083
C	-1.09753796	-0.78451215	0.06952119
C	-0.80316618	-1.47457304	-1.11007417
C	-0.72208617	-1.31426832	1.30784622

C	-0.14150305	-2.70008405	-1.04306921
H	-1.08698034	-1.07716410	-2.07581418
C	-0.05932712	-2.53962322	1.36046391
H	-0.94155608	-0.79081889	2.22929728
C	0.23462185	-3.24109635	0.18848976
H	0.74616691	-4.19933739	0.23533412
C	1.27059903	1.50356613	0.03825105
H	1.06017011	0.44586623	-0.03281111
C	2.58751429	1.89507792	0.16108526
H	2.80382396	2.95488448	0.26408802
C	3.72317523	1.05589912	0.17841423
C	3.66338720	-0.38137328	0.05556414
C	5.02870948	1.64001608	0.34200726
C	4.77362541	-1.14611114	0.04808422
H	2.69588132	-0.86316006	-0.02272975
C	6.16487473	0.91107018	0.35128691
H	5.08924630	2.71528520	0.48085913
C	6.15775254	-0.57188979	0.15541724
H	4.70224734	-2.22602114	-0.02329809
H	7.13896040	1.36010407	0.49828811
O	-7.69396150	-2.65848543	-0.05761311
O	0.27684490	-3.01448836	2.59632721
O	0.11552627	-3.33703248	-2.22508397
O	-0.75332932	5.98603240	-0.12179120
O	6.89982256	-1.17325422	1.19323596
O	-3.46380853	2.05935941	-0.08777516
H	-8.50644384	-2.15727519	-0.18480192
H	0.55552922	-4.17746332	-2.06051990
H	0.17774376	6.23021225	-0.10171475
H	0.75089193	-3.84870243	2.51633201
H	7.05367978	-2.09446926	0.94371989
O	6.88303864	-0.73789322	-1.10261700
O	7.08009843	-2.17161008	-1.32225202
H	6.53721231	-2.30620419	-2.11152729

Cartesian coordinates RAF 7a

C	-1.58315016	2.63559110	0.36408294
C	-0.53207979	1.67708335	0.26944277
C	0.78540487	2.14965504	-0.03031709
C	0.96146532	3.54134940	-0.12506787
C	-0.09526990	4.43612727	0.03661483
C	-1.40857935	3.99092940	0.27216115
C	-2.60739813	0.62696497	0.68434789
C	-1.11175695	0.40603029	0.47097781
H	1.93612408	3.93274054	-0.39649399
H	-2.22848234	4.68960847	0.36521803
C	-3.59022527	-0.07924112	-0.22763618
C	-4.83893858	-0.49685193	0.23286127
C	-3.29214343	-0.22593699	-1.58690520
C	-5.76225657	-1.06523909	-0.63992823
H	-5.09000938	-0.38304707	1.27849435
C	-4.20714328	-0.78776606	-2.46751594
H	-2.33124592	0.10533114	-1.96547596
C	-5.44816448	-1.21327114	-1.99121197
H	-6.72810348	-1.39348615	-0.26677677
H	-3.97706654	-0.90301399	-3.51961715
C	-0.44877088	-0.87085694	0.76322811
C	-0.86503282	-2.07503417	0.17185719
C	0.60274506	-0.89260295	1.70027190
C	-0.22622909	-3.26598832	0.50253000
H	-1.67846292	-2.10206031	-0.53832514
C	1.22339906	-2.09435337	2.02499305
H	0.93532026	0.01559274	2.18448443
C	0.81976594	-3.29148302	1.42841185
H	1.30858813	-4.22810135	1.68413517
C	1.87685517	1.22595287	-0.31261993
H	1.57437111	0.22630900	-0.60125119
C	3.19512513	1.50687931	-0.23978926
H	3.50034504	2.47934623	0.13934877
C	4.30067642	0.61528815	-0.58385396
C	4.13209219	-0.59973616	-1.27091812
C	5.61311855	0.97480799	-0.22558614
C	5.21405964	-1.41623919	-1.57068010
H	3.14432306	-0.90934295	-1.59074914
C	6.70298873	0.16710200	-0.51858312
H	5.77810226	1.90927892	0.30089018
C	6.50597763	-1.03715883	-1.19383022
H	5.05727818	-2.34649099	-2.10952118
H	7.70807360	0.45475429	-0.23493988
O	-6.31382225	-1.76658734	-2.89443339
O	2.22665615	-2.04760691	2.95378909
O	-0.66774919	-4.40230121	-0.11941308
O	0.07547920	5.78667228	-0.06431885
O	7.60856658	-1.79987629	-1.46657717
O	-2.79211432	2.04572410	0.54605324
H	-7.12965958	-2.01969916	-2.44980226
H	-0.17190005	-5.16518324	0.19488698
H	1.00502190	5.98677325	-0.21858090
H	2.59449202	-2.92696245	3.08867848
H	7.34747635	-2.58933096	-1.95219837
O	-2.95669744	0.47397295	2.09530219
O	-2.93160809	-0.92429908	2.46672800
H	-2.04350103	-1.01061318	2.84651004

Cartesian coordinates RAF 7a

C	-1.67303039	2.73267343	-0.15142513
C	-0.72385098	1.70811695	-0.02957095
C	0.67115921	2.06132319	-0.00960880
C	0.96484623	3.45584335	-0.10253127
C	-0.02443000	4.42206651	-0.21117876
C	-1.38636131	4.08297449	-0.24492228
C	-2.81153301	0.83989531	-0.06193226
C	-1.48215086	0.48359127	0.02296206
H	1.99867813	3.77362223	-0.03773193
H	-2.15289837	4.84059861	-0.33198781
C	-4.07462407	0.11287986	-0.06632178
C	-5.27529920	0.80084891	-0.31619822
C	-4.15257717	-1.27155102	0.17909898
C	-6.49611059	0.13846081	-0.32617798
H	-5.24880436	1.86551438	-0.50541878
C	-5.36895564	-1.93626432	0.17031676
H	-3.25471843	-1.83613922	0.38501085
C	-6.54890237	-1.23485734	-0.08358408
H	-7.40921651	0.69254697	-0.52360925
H	-5.42128068	-3.00096524	0.36267322
C	-0.93923194	-0.89674324	0.12109375
C	-0.72636615	-1.63928422	-1.04321791
C	-0.63027497	-1.44202705	1.37130992
C	-0.20902978	-2.93027245	-0.95137814

H	-0.95682602	-1.22868819	-2.01752319
C	-0.11432187	-2.73425726	1.44787235
H	-0.78814707	-0.87787383	2.28138631
C	0.09748503	-3.48782947	0.29147083
H	0.49847015	-4.49625239	0.35748728
C	1.68842891	1.09967107	0.08835514
H	1.42200510	0.06043575	0.21146981
C	3.15044405	1.41844586	-0.00112503
H	3.34277405	2.12248338	-0.81950400
C	4.02240751	0.19460017	-0.18761186
C	4.17991553	-0.73209397	0.84714292
C	4.68854641	-0.03809177	-1.39380525
C	4.97917056	-1.86015593	0.68232009
H	3.67798614	-0.56647684	1.79426701
C	5.48210061	-1.16566688	-1.57654391
H	4.58786515	0.67454012	-2.20593516
C	5.63008821	-2.07967294	-0.53375781
H	5.09677649	-2.56779397	1.49831299
H	5.99816855	-1.34480132	-2.51195836
O	-7.71865079	-1.94271393	-0.07930727
O	0.16941292	-3.22290747	2.69433242
O	-0.02241119	-3.61332439	-2.12055717
O	0.26660796	5.75871320	-0.28479896
O	6.42713074	-3.17100523	-0.75768181
O	-2.92993801	2.21175823	-0.16998179
H	-8.45804265	-1.35306019	-0.26118019
H	0.36793588	-4.47518955	-1.94261127
H	1.21870103	5.88503840	-0.21203127
H	0.49111300	-4.12789529	2.62837418
H	6.48227842	-3.70355652	0.04257019
O	3.47566422	2.11910035	1.23556326
O	4.78607113	2.73511029	1.08032382
H	5.37084533	2.05033429	1.43847998

Cartesian coordinates RAF 8b

C	1.10807599	2.80214911	-0.32932023
C	0.31199814	1.76847914	0.19848222
C	-1.02055008	2.07484208	0.54376719
C	-1.44509681	3.39666546	0.39302523
C	-0.60323296	4.40021149	-0.10551212
C	0.70170226	4.11326354	-0.49492202
C	2.37736848	0.99209621	-0.30732286
C	1.16973943	0.59430309	0.20592615
H	-2.46006089	3.66236801	0.66830694
H	1.35203835	4.87864434	-0.89642732
C	3.65312222	0.32955783	-0.56428710
C	4.58656972	0.92333916	-1.42964801
C	4.00148331	-0.89222512	0.03957611
C	5.80970261	0.31834986	-1.69259724
H	4.34915752	1.86767607	-1.90157410
C	5.22088867	-1.49971214	-0.21922120
H	3.31872136	-1.36835235	0.72929409
C	6.13119683	-0.89842390	-1.08991622
H	6.51392957	0.79527932	-2.36817032
H	5.48620650	-2.43751794	0.25348596
C	0.82913238	-0.77025294	0.68451300
C	0.77545105	-1.02985407	2.05707707
C	0.56977732	-1.79214236	-0.23599030
C	0.47104237	-2.31454403	2.50410802
H	0.95907026	-0.24776925	2.78202503
C	0.26214806	-3.07019009	0.22626190
H	0.61287404	-1.60721608	-1.30154319
C	0.21282133	-3.34257244	1.59402634
H	-0.02765692	-4.34206761	1.94786600
C	-1.98736326	1.02578099	1.06866710
H	-1.53228723	0.04196587	0.98526295
C	-3.33135123	1.05440895	0.41421296
H	-3.93327818	1.93916685	0.60203808
C	-3.88004507	0.06997681	-0.43467201
C	-3.20767510	-1.13182018	-0.79806015
C	-5.18041719	0.26233803	-0.99286617
C	-3.78984098	-2.05642142	-1.64722029
H	-2.21689283	-1.33836494	-0.41548299
C	-5.76099713	-0.66091593	-1.83672713
H	-5.72419338	1.16745180	-0.74339402
C	-5.06698540	-1.83103795	-2.17399508
H	-3.24636431	-2.95997252	-1.90940434
H	-6.74845722	-0.49886016	-2.25221224
O	7.31761487	-1.54293428	-1.30824205
O	0.00421596	-4.03329744	-0.71438505
O	0.44963238	-2.51812910	3.85361213
O	-1.02469089	5.69736727	-0.24344482
O	-5.68511047	-2.71016011	-3.01855721
O	2.34527547	2.33524809	-0.64379387
H	7.86158368	-1.02831416	-1.91375841
H	0.16553405	-3.41673504	4.04991308
H	-1.93012586	5.77937070	0.07289999
H	-0.13759878	-4.88225915	-0.28286694
H	-5.10378809	-3.45808548	-3.19289306
O	-2.09487726	1.32406233	2.49251639
O	-2.73770696	0.19372491	3.14120409
H	-3.66521339	0.47264313	3.15117326

Cartesian coordinates RAF 10b

C	1.44977791	2.57591525	-0.26972588
C	0.55990181	1.51687918	-0.09591391
C	-0.84000221	1.76536123	-0.09252876
C	-1.28130693	3.21365745	-0.09646319
C	-0.21121687	4.25029822	-0.34272905
C	1.10496126	3.94056548	-0.41817915
C	2.69572733	0.75643824	-0.14138081
C	1.39564520	0.33054422	-0.02141104
H	-2.09360829	3.35893936	-0.82154609
H	1.85621726	4.70202932	-0.58206322
C	4.00357250	0.11132778	-0.12483820
C	5.13888525	0.81745926	-0.55756020
C	4.18642758	-1.20877895	0.32733793
C	6.39823237	0.23019210	-0.55032899
H	5.03178824	1.83659696	-0.90469783
C	5.44101635	-1.79936828	0.33509091
H	3.34112725	-1.77819325	0.68708797
C	6.55473231	-1.08327504	-0.10630324
H	7.25979644	0.79644086	-0.89229286
H	5.57496857	-2.81465198	0.68816697
C	0.94373629	-1.07218984	0.16263786
C	0.52189418	-1.50570401	1.42382706
C	0.93712211	-1.95427539	-0.92216703
C	0.10427491	-2.82381310	1.59498891
H	0.51731680	-0.83355418	2.27201141

C	0.51822698	-3.27056200	-0.73641206
H	1.25794587	-1.63329817	-1.90454808
C	0.10001397	-3.71576411	0.51947425
H	-0.22247617	-4.74468047	0.65929823
C	-1.82011097	0.74714483	-0.13640708
H	-1.43761213	-0.26461826	-0.18789020
C	-3.18292707	0.92239828	-0.14040713
H	-3.57955812	1.92694392	-0.03795894
C	-4.18577034	-0.11849602	-0.24914882
C	-3.88742021	-1.49090134	-0.37897505
C	-5.55177050	0.23869228	-0.22792775
C	-4.89188436	-2.44218793	-0.48102318
H	-2.85678825	-1.82358925	-0.40134178
C	-6.56227732	-0.70420897	-0.33037224
H	-5.81684438	1.28604095	-0.12789420
C	-6.23557228	-2.05509636	-0.45811509
H	-4.63381837	-3.49286618	-0.58210291
H	-7.60573178	-0.41348481	-0.31359890
O	7.76730864	-1.71658220	-0.07594117
O	0.53607296	-4.09305320	-1.82952503
O	-0.28915197	-3.19797528	2.84991000
O	-0.63071525	5.53191524	-0.49868500
O	-7.26513742	-2.95131518	-0.55807985
O	2.72776319	2.13343206	-0.30032396
H	8.45390660	-1.12100432	-0.39401321
H	-0.55631505	-4.12286757	2.85201719
H	-1.52327587	5.60709440	-0.12474084
H	0.24474977	-4.97706655	-1.58364018
H	-6.91327636	-3.84262112	-0.65207390
O	-1.88846426	3.42917547	1.22275823
O	-2.71479306	4.65903261	1.11576123
H	-2.66490805	4.95785212	2.03460820

Cartesian coordinates RAF 11a

C	1.57483308	3.04219026	-0.24209012
C	0.57828701	2.05134238	-0.20637278
C	-0.77986121	2.45502418	-0.26369008
C	-1.02388230	3.83250114	-0.38170984
C	0.00460384	4.78108628	-0.41620214
C	1.34321590	4.40173710	-0.34420281
C	2.63278329	1.10824533	-0.04327823
C	1.29677425	0.79675308	-0.07048906
H	-2.04778207	4.17691932	-0.48400403
H	2.14083525	5.13126033	-0.37490197
C	3.86852541	0.34213108	0.09115025
C	5.07117913	1.00692112	0.38510292
C	3.91397644	-1.05552499	-0.06737908
C	6.26373827	0.30812986	0.52555080
H	5.06905925	2.08191136	0.50647025
C	5.10171531	-1.75734526	0.07298307
H	3.01775497	-1.60478187	-0.31766087
C	6.28393538	-1.07891303	0.37285088
H	7.17982453	0.84453302	0.75547408
H	5.12833663	-2.83254029	-0.05507624
C	0.70871121	-0.56159888	0.07379483
C	0.50787310	-1.08893089	1.37342399
C	0.34415677	-1.28907102	-1.02669118
C	-0.06450724	-2.37770609	1.55122013
H	0.78992881	-0.51853391	2.24861119
C	-0.26286721	-2.65353233	-0.91719223
H	0.47109211	-0.90635998	-2.03137705
C	-0.43754809	-3.14107814	0.48939900
H	-0.84593905	-4.13789621	0.61802981
C	-1.87793840	1.48707591	-0.21598105
H	-1.62739837	0.47762586	-0.51732103
C	-3.13785810	1.75256996	0.18104900
H	-3.36586417	2.74954942	0.55116298
C	-4.27132931	0.82773323	0.20395497
C	-4.22701124	-0.47178427	-0.33168883
C	-5.48335635	1.24698290	0.78172392
C	-5.33600959	-1.30566813	-0.28188690
H	-3.32348025	-0.84441607	-0.79982125
C	-6.59839155	0.42158228	0.83604502
H	-5.54964162	2.24634208	1.19977712
C	-6.52712831	-0.86393795	0.30195224
H	-5.27997131	-2.30439441	-0.70635796
H	-7.52565985	0.75734313	1.28420894
O	7.42515374	-1.82153234	0.49937504
O	-1.48739924	-2.64582433	-1.63329500
O	-0.19732808	-2.77253635	2.85528115
O	-0.25416595	6.12201032	-0.53840398
O	-7.64931481	-1.64490091	0.37172691
O	2.80866223	2.47710516	-0.14777804
H	8.16996861	-1.24412429	0.69748101
H	-0.58436514	-3.65383238	2.89300302
H	-1.20537423	6.26500036	-0.57641902
H	-1.86972927	-3.52985303	-1.55749935
H	-7.47730152	-2.49835798	-0.03941894
O	0.69580405	-3.49269843	-1.62255799
O	0.13338495	-4.84460343	-1.68870991
H	0.13760090	-4.97303951	-2.64754945

Cartesian coordinates RAF 12b

C	1.57938824	2.35668823	-0.27390902
C	0.54045507	1.46585220	-0.17997477
C	-0.82667488	1.94797607	-0.23661526
C	-0.96767627	3.37075324	-0.40998325
C	0.07438486	4.23699629	-0.48240486
C	1.51429309	3.83019128	-0.34603906
C	2.52332033	0.37243495	-0.13668013
C	1.16653791	0.15659995	-0.08891712
H	-1.96773925	3.77931829	-0.51330405
H	2.10517240	4.23761013	-1.17827135
C	3.70853449	-0.48262500	-0.10291386
C	4.94249540	0.01373215	-0.55332890
C	3.66889012	-1.80181202	0.38161617
C	6.08632472	-0.77528118	-0.53167708
H	5.00523518	1.02801200	-0.92557281
C	4.80724828	-2.59439730	0.40351988
H	2.74158530	-2.21216622	0.75661922
C	6.02276736	-2.08537641	-0.05537893
H	7.02873278	-0.36961285	-0.88817109
H	4.77120548	-3.60877240	0.78182023
C	0.50311897	-1.17019293	0.00632896
C	0.02097606	-1.62583518	1.23849014
C	0.37600584	-1.96616336	-1.13492803
C	-0.57791289	-2.88108913	1.32230335
H	0.11197020	-1.02160631	2.13190908
C	-0.22636615	-3.21966044	-1.03737897

H	0.74362527	-1.62835618	-2.09491801
C	-0.70681094	-3.68655141	0.18788279
H	-1.17047710	-4.66748833	0.25933178
C	-1.93874192	1.07605706	-0.16489904
H	-1.70088310	0.02370079	-0.08211402
C	-3.26972007	1.42715029	-0.18704087
H	-3.53510617	2.47839000	-0.24557105
C	-4.40339131	0.52796914	-0.13219694
C	-4.29333514	-0.87753410	-0.07375620
C	-5.70972621	1.06587093	-0.13849098
C	-5.41779235	-1.68817121	-0.02301974
H	-3.31718251	-1.34671324	-0.06915976
C	-6.83848033	0.26402399	-0.08795296
H	-5.83230535	2.14316839	-0.18274713
C	-6.69730748	-1.12345124	-0.02947888
H	-5.30430658	-2.76799231	0.02021087
H	-7.83321978	0.69295390	-0.09274784
O	7.11204168	-2.91161630	-0.01039992
O	-0.32053113	-3.95837653	-2.18386635
O	-1.02195716	-3.28207848	2.55215625
O	-0.05453701	5.57645768	-0.68567983
O	-7.83893754	-1.87651597	0.01810580
O	2.77722539	1.72078122	-0.25188202
H	7.88757581	-2.44745430	-0.34272588
H	-1.40005589	-4.16571823	2.49689322
H	-0.98714999	5.82042744	-0.68876017
H	-0.73819380	-4.80593856	-1.99939093
H	-7.61178080	-2.81142347	0.05533395
O	2.18349434	4.50124359	0.76120507
O	1.52353623	4.14632124	2.00357196
H	2.15581731	3.51498420	2.37660011

Cartesian coordinates Vam3

C	-1.58624300	2.77765700	0.05608100
C	-0.58276300	1.79388800	0.07957000
C	0.77106700	2.20823400	0.13819700
C	1.00847400	3.59168800	0.16042100
C	-0.02562700	4.53391500	0.11756600
C	-1.36191300	4.14240200	0.06815000
C	-2.62657400	0.82555900	-0.00982700
C	-1.28841600	0.52608000	0.03167800
H	2.02909800	3.94798700	0.25548200
H	-2.16339400	4.86820500	0.05085100
C	-3.85664500	0.04190700	-0.07255300
C	-5.09353400	0.66188800	0.17167800
C	-3.86171100	-1.32995900	-0.38569100
C	-6.28181800	-0.05668200	0.11810100
H	-5.12219100	1.71723100	0.40798500
C	-5.04481600	-2.05114200	-0.44032500
H	-2.93257400	-1.83903800	-0.59930000
C	-6.26253900	-1.41825300	-0.18587800
H	-7.22518400	0.44489900	0.31394600
H	-5.04171000	-3.10602300	-0.68650700
C	-0.68342500	-0.83101300	0.02613200
C	-0.14408000	-1.34381800	-1.15768200
C	-0.64700900	-1.58829500	1.20091000
C	0.42265800	-2.61651900	-1.16233300
H	-0.16017500	-0.76785000	-2.07371400
C	-0.07820800	-2.86072400	1.18157600
H	-1.06093400	-1.20545000	2.12462900
C	0.45865400	-3.38471500	0.00399000
H	0.89804100	-4.37919100	-0.00569200
C	1.87106000	1.24352800	0.20238300
H	1.60821900	0.26043100	0.57434100
C	3.14314300	1.47780700	-0.17373300
H	3.38275900	2.43603100	-0.62890500
C	4.27420400	0.55446600	-0.07320500
C	4.23462000	-0.64309800	0.66120500
C	5.47802200	0.86638100	-0.73032400
C	5.33441700	-1.48862700	0.72309500
H	3.33780400	-0.91607300	1.20446300
C	6.58436700	0.02950100	-0.67692700
H	5.54313100	1.78684800	-1.30141500
C	6.51507900	-1.15676400	0.05230500
H	5.28147600	-2.40438000	1.30554200
H	7.50496400	0.28134700	-1.18929100
O	-7.39746000	-2.17923200	-0.25294800
O	-0.07037300	-3.56116700	2.35749400
O	0.92751700	-3.07221200	-2.34824200
O	0.22471100	5.88216900	0.14633300
O	7.63046400	-1.94998600	0.08369500
O	-2.81550400	2.19815400	0.00924900
H	-8.16769100	-1.63244800	-0.06555400
H	1.31851400	-3.94388900	-2.22959700
H	1.17496200	6.03310100	0.17543600
H	0.31146900	-4.43405200	2.21929000
H	7.46591300	-2.72125800	0.63601700

Cartesian coordinates Vam3 radical cation

C	1.58547600	2.78824700	0.00006300
C	0.57507100	1.79122800	0.00004200
C	-0.80449600	2.21345300	-0.00000800
C	-1.03212600	3.60462600	-0.00009500
C	0.00457800	4.53271400	-0.00008200
C	1.36402400	4.13377200	-0.00000400
C	2.63345600	0.84828200	0.00011000
C	1.25676700	0.54737600	0.00002700
H	-2.04835200	3.97948400	-0.00019000
H	2.15789600	4.86808600	-0.00000900
C	3.83290100	0.05996400	0.00011500
C	5.09129100	0.71058300	0.00006100
C	3.81631300	-1.35694300	0.00017600
C	6.26654200	-0.01142600	0.00007400
H	5.13302600	1.79106600	-0.00000100
C	4.98874900	-2.08028500	0.00019000
H	2.87685900	-1.88827200	0.00023200
C	6.22515600	-1.41530500	0.00015000
H	7.22120600	0.50428400	0.00000600
H	4.97940000	-3.16282500	0.00025000
C	0.66490100	-0.81655600	-0.00008500
C	0.39245500	-1.45002400	1.21491000
C	0.39252200	-1.44985600	-1.21517400
C	-0.14920000	-2.73792500	1.20687500
H	0.60199100	-0.97224500	2.16320400
C	-0.14913900	-2.73776300	-1.20734900
H	0.60211300	-0.97195300	-2.16339400
C	-0.42279400	-3.38667100	-0.00029100
H	-0.83704800	-4.39132900	-0.00037000

C	-1.86358200	1.24342900	0.00005300
H	-1.55105900	0.20920800	0.00015100
C	-3.20322800	1.51588100	0.00002400
H	-3.52646500	2.55283600	-0.00000200
C	-4.27909000	0.56084800	0.00004500
C	-4.08418900	-0.84297100	0.00006600
C	-5.60992600	1.03644300	0.00007900
C	-5.15215700	-1.71173500	0.00012400
H	-3.08154800	-1.25187000	0.00003900
C	-6.68962700	0.17284800	0.00013300
H	-5.78945800	2.10599900	0.00005500
C	-6.46625600	-1.21086600	0.00016700
H	-5.00818500	-2.78490100	0.00014300
H	-7.70261300	0.56161300	0.00015000
O	7.33132500	-2.18100300	0.00015400
O	-0.38807000	-3.30931900	-2.41730000
O	-0.38819000	-3.30966300	2.41672800
O	-0.20119200	5.86552400	-0.00017700
O	-7.46298800	-2.11851700	0.00022800
O	2.81564900	2.20439400	0.00010900
H	8.13212700	-1.64220400	0.00016200
H	-0.72441600	-4.20700800	2.31499500
H	-1.14270400	6.07636500	-0.00025900
H	-0.72425100	-4.20669800	-2.31572100
H	-8.32713700	-1.68915800	0.00025700

Cartesian coordinates RAF TS 4b

C	-2.32824600	2.72425300	-0.06509600
C	-1.23803500	1.83949900	0.01666900
C	0.07395200	2.38452900	0.07658400
C	0.17790000	3.79001100	0.03710700
C	-0.93740500	4.62303100	-0.05369700
C	-2.23263500	4.10104400	-0.10504700
C	-3.18349700	0.68479300	-0.05943600
C	-1.82185100	0.51316600	0.01373700
H	1.15695000	4.25105000	0.10887200
H	-3.09782900	4.74690200	-0.16706500
C	-4.33476800	-0.21008300	-0.09771900
C	-5.63402900	0.32117100	-0.02320600
C	-4.20369000	-1.60685600	-0.21405400
C	-6.75168000	-0.50294100	-0.05649600
H	-5.76755600	1.39110800	0.06252400
C	-5.31620300	-2.43292800	-0.24869800
H	-3.22302900	-2.05483400	-0.28424600
C	-6.59798200	-1.88548900	-0.16838900
H	-7.74501100	-0.06797600	0.00479500
H	-5.20788100	-3.50671800	-0.34068300
C	-1.09116200	-0.78056500	0.06840800
C	-0.59119800	-1.34179200	-1.11015600
C	-0.88553000	-1.41867000	1.29536400
C	0.11258100	-2.54418500	-1.05691200
H	-0.73744500	-0.85642800	-2.06630800
C	-0.18262800	-2.62247000	1.33389600
H	-1.26494200	-0.99571600	2.21646000
C	0.31985400	-3.19342900	0.16262200
H	0.86409600	-4.13378600	0.19892200
C	1.23752300	1.52736900	0.19905600
H	1.02484900	0.48601600	0.39816400
C	2.54355800	1.90502900	0.07799400
H	2.76765300	2.93414400	-0.18945000
C	3.69333700	1.06565400	0.24371800
C	3.62223400	-0.29063000	0.68550100
C	4.98836600	1.60612900	-0.01094600
C	4.74443700	-1.05328200	0.83040700
H	2.66085700	-0.71995200	0.93927200
C	6.12156300	0.85660300	0.12154800
H	5.06636500	2.64615100	-0.31044900
C	6.03643900	-0.54171200	0.46366900
H	4.69828500	-2.07198100	1.19475600
H	7.10181800	1.27907300	-0.06609700
O	-7.65694400	-2.74840800	-0.20631500
O	-0.01401400	-3.20793500	2.55784600
O	0.57278700	-3.04938400	-2.23856200
O	-0.82650100	5.98811200	-0.08336700
O	7.10810000	-1.16777000	1.02578600
O	-3.49709300	2.03221200	-0.10514700
H	-8.48261000	-2.25634100	-0.14562000
H	1.11160800	-3.83173000	-2.08136100
H	0.10163600	6.24095900	-0.04443400
H	0.48640300	-4.02572000	2.46881400
H	7.82997900	-1.16669800	0.37083700
O	6.18298100	-1.25081100	-1.34087500
O	7.52532500	-1.68868400	-1.48676700
H	7.45143700	-2.64967300	-1.38705900

Cartesian coordinates RAF TS 7b

C	-1.70904100	2.72340000	-0.18775500
C	-0.75072500	1.69976600	-0.10640200
C	0.62928200	2.06041600	-0.10892400
C	0.92433000	3.44037300	-0.18654900
C	-0.06941100	4.41407100	-0.25824200
C	-1.42622600	4.07368200	-0.26660400
C	-2.83653500	0.82309700	-0.10104200
C	-1.50755900	0.46877400	-0.05593600
H	1.95836700	3.75634300	-0.11435700
H	-2.19648900	4.83115600	-0.31706800
C	-4.09955900	0.09389300	-0.08462600
C	-5.31323300	0.80148300	-0.03127700
C	-4.16306500	-1.31188900	-0.12649000
C	-6.53357000	0.13804100	-0.01602200
H	-5.29795300	1.88255200	-0.00048800
C	-5.37866500	-1.97797900	-0.11211200
H	-3.25418200	-1.89371000	-0.17559400
C	-6.57223800	-1.25630200	-0.05567000
H	-7.45708400	0.70812000	0.02739700
H	-5.41951900	-3.05985600	-0.14631800
C	-0.95863300	-0.91050000	0.02407200
C	-0.59953200	-1.58146000	-1.14855100
C	-0.79451300	-1.52817600	1.26755200
C	-0.08531300	-2.87444500	-1.07195500
H	-0.71730900	-1.11435900	-2.11761800
C	-0.28062700	-2.82273300	1.32911100
H	-1.06705500	-1.02085200	2.18384400
C	0.07580400	-3.50488800	0.16398100
H	0.47311400	-4.51549500	0.21772500
C	1.67037400	1.07703700	-0.02094700
H	1.37819300	0.08652400	0.30043700
C	3.02033500	1.30759300	-0.30134500
H	3.25923600	2.16052000	-0.92699800

C	4.01406700	0.22707600	-0.34378500
C	3.91919500	-0.91683000	0.46311000
C	5.13706000	0.34416800	-1.18109800
C	4.89359600	-1.90971600	0.41967300
H	3.08420100	-1.03175300	1.14330000
C	6.11084200	-0.64180100	-1.23780400
H	5.24307600	1.22704000	-1.80245400
C	5.98987500	-1.77764600	-0.43443200
H	4.80408400	-2.78444000	1.05768400
H	6.96893900	-0.54797700	-1.89214100
O	-7.74035100	-1.96649400	-0.04323500
O	-0.14487100	-3.38356300	2.56938900
O	0.24269600	-3.48740200	-2.24877800
O	0.22661100	5.75033400	-0.30776900
O	6.97757400	-2.72011300	-0.52185200
O	-2.96349200	2.19769300	-0.18074100
H	-8.48955000	-1.36269900	-0.00309700
H	0.60158300	-4.36424600	-2.07793500
H	1.17941600	5.87171700	-0.23703300
H	0.18631900	-4.28419000	2.49138300
H	6.79810200	-3.43963300	0.09242000
O	3.67268400	2.40793900	1.21422500
O	3.89679200	1.64466700	2.35629600
H	4.78547500	1.27973200	2.21703600

Cartesian coordinates RAF TS 11a

C	1.53771100	3.06959900	-0.23403300
C	0.53652100	2.08185300	-0.20477500
C	-0.81659100	2.50425700	-0.22761400
C	-1.05479800	3.88533900	-0.29371000
C	-0.02044400	4.82848600	-0.31736100
C	1.31436500	4.43365400	-0.28942400
C	2.58855500	1.13159800	-0.09416700
C	1.25190500	0.82022300	-0.09540500
H	-2.07954800	4.23426200	-0.37048100
H	2.11884000	5.15594800	-0.31461300
C	3.81843800	0.34960500	-0.01344400
C	5.00334700	0.95380600	0.43756000
C	3.87091600	-1.00425300	-0.39101500
C	6.18753300	0.23285600	0.52658900
H	4.99343300	1.99751800	0.72381300
C	5.05022500	-1.72848400	-0.30316700
H	2.98651700	-1.49345900	-0.77601000
C	6.21466800	-1.11402200	0.16051200
H	7.09107900	0.71925700	0.88249900
H	5.08789400	-2.76882200	-0.60218200
C	0.70650000	-0.55034100	0.10088700
C	0.05090391	-1.17376638	-0.99189237
C	0.79825665	-1.20145418	1.30296176
C	-0.52970783	-2.46042340	-0.85074743
H	-0.01718755	-0.67256518	-1.94836664
C	0.22329065	-2.57113418	1.52208136
H	1.24526808	-0.72972220	2.17075278
C	-0.48248570	-3.13697881	0.32888211
H	-0.88822674	-4.13525796	0.44130029
C	-1.93095000	1.55520200	-0.21568400
H	-1.71711300	0.57809100	-0.63176800
C	-3.16086500	1.78542200	0.28154900
H	-3.34632200	2.71454700	0.81597200
C	-4.29159000	0.85594000	0.22157800
C	-4.42313400	-0.11586200	-0.78366200
C	-5.30384200	0.91768800	1.19560000
C	-5.48512600	-1.01245200	-0.79252000
H	-3.70804600	-0.14242100	-1.59920900
C	-6.36950900	0.02912500	1.19909000
H	-5.24108500	1.66880200	1.97612900
C	-6.45846400	-0.94924100	0.20733900
H	-5.56583400	-1.75027100	-1.58532800
H	-7.13806600	0.07690800	1.96098500
O	7.34717700	-1.87636000	0.22656200
O	1.19703338	-3.52097092	1.95362733
O	-1.11860738	-2.95559381	-1.98554085
O	-0.26998000	6.17406400	-0.38995200
O	-7.52625800	-1.80145400	0.25410500
O	2.77128000	2.49905900	-0.17725300
H	8.08255500	-1.34309900	0.54643200
H	-1.45012687	-3.84432359	-1.81755200
H	-1.22034700	6.32728600	-0.39874500
H	2.07512628	-3.13786548	1.86052252
H	-7.46610600	-2.44165600	-0.46287500
O	-0.67816418	-2.33708021	2.62559334
O	-1.35156507	-3.58351854	2.97509177
H	-2.27206437	-3.31284260	2.85034993

Cartesian coordinates RAF TS 8b

C	1.68559700	2.74021400	-0.20727200
C	0.70439300	1.74479200	-0.05742100
C	-0.64484700	2.14997600	0.04826000
C	-0.91368900	3.52258600	-0.00469800
C	0.10018100	4.47697900	-0.15533200
C	1.43620500	4.09946500	-0.26300900
C	2.75729500	0.80541500	-0.19406700
C	1.43005000	0.48826000	-0.05362500
H	-1.93591300	3.86268900	0.12427500
H	2.22308100	4.83367300	-0.37047500
C	3.99675400	0.03691700	-0.26092800
C	5.23160000	0.68639300	-0.09758900
C	4.01099300	-1.35014600	-0.49660200
C	6.42802500	-0.01815000	-0.15509200
H	5.25218500	1.75360800	0.07889100
C	5.20220600	-2.05759400	-0.55450400
H	3.08232500	-1.88212700	-0.64751900
C	6.41835400	-1.39503600	-0.38105100
H	7.37012300	0.50585500	-0.02201300
H	5.20654900	-3.12471900	-0.74047500
C	0.85960400	-0.87573600	0.09442000
C	0.90246800	-1.51707600	1.33507500
C	0.28492400	-1.51540900	-1.00957200
C	0.38118100	-2.80367600	1.46293800
H	1.33573800	-1.03167500	2.19950500
C	-0.23341500	-2.80069200	-0.86727000
H	0.24551700	-1.03243800	-1.97739300
C	-0.18830100	-3.45473900	0.36628000
H	-0.58756800	-4.46077400	0.47053400
C	-1.74431500	1.18732400	0.24645000
H	-1.44325700	0.20225900	0.57295400
C	-2.95166100	1.31394000	-0.44050400
H	-3.13363200	2.24378800	-0.97054700
C	-4.02055800	0.35327600	-0.48178900

C	-3.97967100	-0.89787100	0.17656400
C	-5.19026000	0.65331800	-1.22206000
C	-5.04297900	-1.78399500	0.10016800
H	-3.11314700	-1.17133800	0.76341100
C	-6.25277900	-0.22799000	-1.30460800
H	-5.25124600	1.60440900	-1.74049600
C	-6.18442900	-1.45604200	-0.63927700
H	-4.99114900	-2.73693300	0.61944200
H	-7.14177100	0.01292700	-1.87470200
O	7.56157400	-2.14341400	-0.44729100
O	-0.77265900	-3.38544700	-1.98045100
O	0.45703300	-3.38962300	2.69563800
O	-0.17211500	5.82031000	-0.19132500
O	-7.25932600	-2.29100000	-0.74616100
O	2.92099600	2.17779000	-0.28663000
H	8.32949200	-1.57756400	-0.31517800
H	0.06777800	-4.26967400	2.66891600
H	-1.11514700	5.96153300	-0.05938500
H	-1.08750300	-4.27042500	-1.76979300
H	-7.10232500	-3.09484000	-0.23935000
O	-2.21178400	1.73544000	2.06845400
O	-2.93133600	0.72594100	2.71811400
H	-3.84675800	1.04356200	2.68329800

Cartesian coordinates RAF TS 7a

C	-1.42473100	2.69544400	-0.14945000
C	-0.39902500	1.71580300	-0.17508300
C	0.95412700	2.15234000	-0.30666400
C	1.15923800	3.53384300	-0.43589000
C	0.10850200	4.45674600	-0.40542400
C	-1.22280400	4.05049300	-0.25279200
C	-2.43999200	0.75886100	0.19100700
C	-1.05569000	0.46547100	-0.01530700
H	2.16639700	3.90645200	-0.58998900
H	-2.03124700	4.76760000	-0.22139300
C	-3.62202600	-0.07232500	-0.10158900
C	-4.75176400	0.50268200	-0.69697300
C	-3.66549700	-1.43861000	0.22289900
C	-5.87936300	-0.26174000	-0.98092600
H	-4.74759400	1.55620900	-0.94133700
C	-4.78618900	-2.20596100	-0.05864900
H	-2.82021400	-1.90197100	0.71261000
C	-5.89894900	-1.61987500	-0.66630000
H	-6.74384600	0.20333700	-1.44571700
H	-4.81785000	-3.25912000	0.19284600
C	-0.45763600	-0.89079200	0.02276300
C	-0.16599400	-1.55469300	-1.17370600
C	-0.18976400	-1.49728000	1.25348800
C	0.39199800	-2.83078600	-1.13047100
H	-0.37775200	-1.10085400	-2.13321000
C	0.37270900	-2.77232500	1.27840900
H	-0.42447100	-0.99314200	2.18168300
C	0.66655900	-3.44774100	0.09129600
H	1.09986100	-4.44466700	0.11857200
C	2.06431800	1.20599000	-0.30598500
H	1.80053700	0.17538300	-0.50603200
C	3.35484200	1.50734400	-0.05002400
H	3.60665100	2.52579500	0.23553000
C	4.49222800	0.59052400	-0.07247800
C	4.42301300	-0.72138900	-0.57344000
C	5.73518000	1.02603600	0.42307100
C	5.53281900	-1.55481900	-0.56659400
H	3.49388500	-1.09705300	-0.98492000
C	6.85180500	0.20220700	0.43583000
H	5.82264200	2.03527700	0.81213000
C	6.75330600	-1.09737400	-0.06004300
H	5.45585600	-2.56274600	-0.96479700
H	7.80225400	0.54900200	0.82271200
O	-6.97526600	-2.42435200	-0.92023700
O	0.61434000	-3.32461300	2.50519300
O	0.65519200	-3.44062500	-2.32790800
O	0.32516300	5.79863000	-0.53583200
O	7.87873500	-1.87411900	-0.03284300
O	-2.64306700	2.11110200	-0.01050300
H	-7.67694200	-1.90974200	-1.33295300
H	0.98118000	-4.33402400	-2.17813200
H	1.26910600	5.97037200	-0.61989900
H	1.01922400	-4.19229500	2.40500900
H	7.68699900	-2.74027900	-0.40719400
O	-2.41534700	0.70118400	2.14960900
O	-3.66621400	1.10142000	2.63441900
H	-4.16027400	0.26901400	2.70345000

Cartesian coordinates RAF TS 11b

C	1.63327200	2.46068300	-0.43848400
C	0.61956100	1.47286000	-0.36299600
C	-0.75447700	1.88115300	-0.49266300
C	-0.98834400	3.22630500	-0.65344500
C	0.05220700	4.23089000	-0.56613200
C	1.42208000	3.79989100	-0.59495900
C	2.65777300	0.51425900	-0.18411400
C	1.29821700	0.22949200	-0.19271600
H	-1.99790500	3.58159400	-0.82335800
H	2.21639500	4.52916500	-0.67032700
C	3.86592000	-0.27898800	-0.04285000
C	5.12056700	0.31171000	-0.28551500
C	3.84190300	-1.63303600	0.34817500
C	6.29461100	-0.41650800	-0.15424700
H	5.17037900	1.35046000	-0.58318000
C	5.01199200	-2.36182600	0.48249300
H	2.89999500	-2.11825700	0.55949900
C	6.24628200	-1.75852400	0.22927100
H	7.25057500	0.05994500	-0.35079000
H	4.98800300	-3.40054400	0.78845700
C	0.67501600	-1.11320100	-0.05834000
C	0.13381500	-1.50477600	1.16853400
C	0.62161500	-1.97156800	-1.16070800
C	-0.45720800	-2.76153900	1.28914600
H	0.15854500	-0.84647200	2.02697600
C	0.02798200	-3.22525300	-1.02509100
H	1.03556800	-1.68128800	-2.11766100
C	-0.51217900	-3.63037300	0.19703400
H	-0.97096100	-4.61091600	0.29728100
C	-1.84925900	0.90407400	-0.50549700
H	-1.61503900	-0.07194200	-0.91560600
C	-3.08455400	1.14263500	-0.02859200
H	-3.26578900	2.09005400	0.47363300
C	-4.23167800	0.23320700	-0.06711400
C	-4.28204500	-0.91283200	-0.87775300

C	-5.35468300	0.50675300	0.73369600
C	-5.38932700	-1.75134200	-0.87426200
H	-3.45310400	-1.14760400	-1.53500300
C	-6.46718800	-0.32356500	0.74737500
H	-5.34882900	1.38936700	1.36477200
C	-6.48687300	-1.46095300	-0.05889500
H	-5.40890000	-2.62683100	-1.51759100
H	-7.32517700	-0.10339700	1.37089400
O	7.36503500	-2.52678200	0.37597000
O	0.00050700	-4.02724900	-2.13308500
O	-0.96450700	-3.09714100	2.51194200
O	-0.16397300	5.46096500	-1.13429200
O	-7.60569600	-2.24901200	-0.01957300
O	2.86245000	1.86810600	-0.33837600
H	8.15033400	-2.00470300	0.17965000
H	-1.39063000	-3.95950900	2.47121800
H	-0.99227000	5.81569200	-0.78616500
H	-0.41205500	-4.87085700	-1.92090400
H	-7.50956000	-2.98110000	-0.63748500
O	-0.17584700	4.85059900	1.15947100
O	-0.18089700	3.83418300	2.13358100
H	0.73386800	3.84002700	2.45249100

Cartesian coordinates RAF TS 10b

C	1.48872700	2.55946900	-0.36965400
C	0.57477100	1.49881300	-0.24533700
C	-0.81071000	1.77262500	-0.31239200
C	-1.19509300	3.16484000	-0.37330900
C	-0.19173100	4.19292500	-0.53583100
C	1.15335000	3.90578800	-0.52186100
C	2.70098300	0.72178100	-0.15721100
C	1.39439300	0.30686200	-0.11205900
H	-2.15084000	3.38156100	-0.83594900
H	1.89977300	4.68143500	-0.62690700
C	3.99886900	0.06425900	-0.05905900
C	5.16576400	0.76199900	-0.41419600
C	4.13949000	-1.25926200	0.39856300
C	6.41632700	0.16263600	-0.32916000
H	5.09057100	1.78357500	-0.76226300
C	5.38511100	-1.86138800	0.48501300
H	3.26747400	-1.82180200	0.70057200
C	6.53155900	-1.15393900	0.11874500
H	7.30332200	0.72179700	-0.61258500
H	5.48691000	-2.87894800	0.84205500
C	0.91663000	-1.08946800	0.05688900
C	0.41919500	-1.50573900	1.29568800
C	0.95724800	-1.97967100	-1.02062900
C	-0.02836900	-2.81592800	1.45270200
H	0.37462500	-0.82621100	2.13679600
C	0.50797400	-3.28788600	-0.84862700
H	1.33834800	-1.67195900	-1.98561400
C	0.01390700	-3.71631400	0.38512100
H	-0.33220100	-4.73889600	0.51414600
C	-1.81331600	0.73928500	-0.33660000
H	-1.44548600	-0.26767400	-0.48936900
C	-3.14741100	0.92984300	-0.16499600
H	-3.49703300	1.92620100	0.08651700
C	-4.18516600	-0.08984600	-0.22331300
C	-3.95844800	-1.41358500	-0.64468700
C	-5.49939200	0.24910500	0.15521800
C	-4.98512300	-2.34630300	-0.67592800
H	-2.96924600	-1.71986600	-0.96296700
C	-6.53307600	-0.67510200	0.12956300
H	-5.70551600	1.26248200	0.48308600
C	-6.27834100	-1.98191500	-0.28714900
H	-4.78666800	-3.36025300	-1.01209500
H	-7.53857600	-0.40251700	0.42622300
O	7.73256500	-1.79868400	0.22450800
O	0.57277200	-4.11940200	-1.93338000
O	-0.49598000	-3.17321700	2.68604200
O	-0.60195100	5.47804600	-0.69612700
O	-7.32738200	-2.85916000	-0.30124900
O	2.76113000	2.09996400	-0.32191100
H	8.44468200	-1.20956100	-0.04618500
H	-0.79416700	-4.08854400	2.67652000
H	-1.48757100	5.56036500	-0.30438700
H	0.25817300	-4.99784900	-1.69624000
H	-7.03063200	-3.71845600	-0.61869400
O	-1.88050300	3.50995600	1.34065100
O	-2.36010100	4.86154000	1.37616800
H	-1.75906900	5.26946200	2.01670200

Cartesian coordinates RAF TS 12b

C	1.57715300	2.39060800	-0.41610200
C	0.54425600	1.46508100	-0.34061200
C	-0.81123200	1.93368800	-0.40574100
C	-0.98060200	3.32754600	-0.61451400
C	0.07547100	4.20926100	-0.70025100
C	1.42897600	3.79570300	-0.45821500
C	2.54914700	0.41931500	-0.22717500
C	1.19570100	0.17350400	-0.21724800
H	-1.98324400	3.71919600	-0.74807100
H	2.23536000	4.42019900	-0.82218300
C	3.75255600	-0.40512300	-0.14326400
C	5.00546800	0.20758900	0.02842100
C	3.71661700	-1.80858100	-0.23297400
C	6.16950100	-0.54603600	0.11326400
H	5.06651000	1.28549900	0.09790600
C	4.87582400	-2.56509300	-0.14812100
H	2.77503000	-2.31819200	-0.37807000
C	6.10999400	-1.93754100	0.02667000
H	7.12534100	-0.04827200	0.24858700
H	4.84071500	-3.64528800	-0.22070600
C	0.54821200	-1.16011700	-0.10262100
C	0.33890500	-1.72715600	1.15711100
C	0.15573600	-1.84689200	-1.25668200
C	-0.25420000	-2.98494500	1.25760800
H	0.63568300	-1.20876200	2.05936900
C	-0.43531800	-3.10379100	-1.14235100
H	0.31418800	-1.42299000	-2.23988600
C	-0.64484200	-3.68201000	0.11214100
H	-1.10137100	-4.66524400	0.19547600
C	-1.92688300	1.03171700	-0.27907000
H	-1.67233200	-0.01953300	-0.24777500
C	-3.23771700	1.37758900	-0.17330400
H	-3.50185400	2.43109400	-0.14771300
C	-4.37457900	0.47612900	-0.06102200
C	-4.27502000	-0.92574800	-0.14834300
C	-5.65977400	1.01695600	0.14465900

C	-5.39434100	-1.73644800	-0.02844900
H	-3.31253700	-1.39326900	-0.31716500
C	-6.78540900	0.21632100	0.26535800
H	-5.77148900	2.09407500	0.21377900
C	-6.65627700	-1.17015500	0.17993600
H	-5.29169600	-2.81562100	-0.10121200
H	-7.76676500	0.64610200	0.42505300
O	7.21917300	-2.73333600	0.10326400
O	-0.79122900	-3.73570200	-2.30196900
O	-0.42843000	-3.49380700	2.51374900
O	-0.07839800	5.53441300	-0.96752600
O	-7.79179300	-1.92151100	0.30246400
O	2.78365000	1.77335900	-0.34172400
H	8.00456400	-2.18843900	0.22036100
H	-0.83925900	-4.36329500	2.46750600
H	-1.01540500	5.75553000	-1.00457900
H	-1.15352700	-4.60639500	-2.10809400
H	-7.57620500	-2.85651200	0.22190400
O	1.78059000	4.42880800	1.28502700
O	0.92186400	3.83422000	2.22247700
H	1.42093400	3.06033400	2.52475400

Cartesian coordinates RAF TS 2b

C	-1.74816100	2.79186400	-0.05850400
C	-0.84991600	1.71012700	-0.04387700
C	0.54363500	1.98815500	-0.04246500
C	0.91932300	3.34532800	-0.06053300
C	-0.01452800	4.38282400	-0.08267500
C	-1.38709400	4.12491700	-0.08181200
C	-2.98522800	0.95925200	-0.03612300
C	-1.68238100	0.52327100	-0.03219000
H	1.97219700	3.60202000	-0.01615400
H	-2.11148400	4.92787700	-0.08802100
C	-4.28966500	0.30551300	-0.02818700
C	-5.44749400	1.06081600	0.22451000
C	-4.44631200	-1.07035400	-0.27935600
C	-6.70451400	0.46929100	0.23554400
H	-5.35937100	2.12200600	0.41532000
C	-5.69869400	-1.66487700	-0.27034600
H	-3.58258000	-1.68314700	-0.49453800
C	-6.83585400	-0.89808000	-0.01036900
H	-7.58401000	1.07412900	0.43642600
H	-5.81276200	-2.72351400	-0.46927600
C	-1.22730800	-0.89151000	-0.00438600
C	-0.83080400	-1.51929800	-1.18952100
C	-1.19343200	-1.58922300	1.20643800
C	-0.40827400	-2.84679300	-1.15727000
H	-0.85087300	-0.99271900	-2.13481000
C	-0.77061200	-2.91767300	1.22411200
H	-1.49878600	-1.11745300	2.13124100
C	-0.37512900	-3.55550100	0.04638300
H	-0.04898300	-4.59252400	0.06467600
C	1.53056900	0.92271800	0.00112600
H	1.15138900	-0.05330600	0.27491600
C	2.85586700	1.05095100	-0.28496600
H	3.22460700	2.01330300	-0.62584400
C	3.85899200	0.02706000	-0.20078100
C	3.63549000	-1.26244800	0.31566500
C	5.21377600	0.37011100	-0.59265300
C	4.64218100	-2.20737700	0.35342900
H	2.65469800	-1.53197600	0.68835900
C	6.20303600	-0.66256900	-0.66455200
H	5.32818600	1.20063200	-1.27935000
C	5.93258300	-1.90619200	-0.15715500
H	4.44722600	-3.19260300	0.76728500
H	7.18079500	-0.44930600	-1.07760500
O	-8.04383400	-1.53814000	-0.01389700
O	-0.76188600	-3.55470600	2.43412000
O	-0.03943000	-3.41440600	-2.34475900
O	0.36316700	5.69920400	-0.08902900
O	6.92382800	-2.85053000	-0.17061500
O	-3.03128700	2.34211300	-0.04985700
H	-8.74828700	-0.90801800	0.17098700
H	0.23774200	-4.32586800	-2.20591000
H	1.32396000	5.76197000	-0.07351400
H	-0.47989000	-4.46890100	2.32621300
H	6.57950600	-3.69093400	0.14826500
O	5.65820500	1.43523300	0.85972300
O	6.90777000	2.04556900	0.61997600
H	7.52414500	1.47182000	1.09912000
